# Machine learning–based prediction of major amputation risk after initial limb-preserving surgery in diabetic foot

**DOI:** 10.3389/fendo.2026.1889329

**Published:** 2026-08-03

**Authors:** Kerim Bora Yilmaz, Gokalp Tulum, Ferhat Cuce, Zehra Simsek, Necibe Sare Mert, Sema Horasan Hatipoglu, Cansu Bozkurt, Muhammet Ikbal Isik, Ali Murat Basak, Onur Osman

**Affiliations:** 1 Department of General Surgery, University of Health Sciences Gulhane Training and Research Hospital, Ankara, Türkiye; 2 Department of Electrical and Electronics Engineering, Engineering Faculty, Istanbul Topkapi University, Istanbul, Türkiye; 3 Department of Radiology, University of Health Sciences, Gulhane Training, and Research Hospital, Ankara, Türkiye; 4 Department of Orthopedics and Traumatology, University of Health Sciences, Gulhane Training and Research Hospital, Ankara, Türkiye

**Keywords:** amputation prediction, clinical decision support, diabetic foot, explainable artificial intelligence, limb-preserving surgery, machine learning, temporal validation, web-based deployment

## Abstract

**Background:**

Accurate preoperative prediction of whether an initially limb-preserving strategy in diabetic foot management will culminate in minor or major amputation remains a clinical challenge. This study aimed to develop and evaluate using two temporally separated cohorts a machine-learning framework using routinely available baseline clinical, laboratory, and selected imaging and vascular variables.

**Methods:**

Two temporally separated cohorts were used, with Dataset 1 for model development and Dataset 2 for temporally separated evaluation. A 20-repetition stratified outer-split workflow was implemented, incorporating two-step feature selection, Optuna-based hyperparameter optimization, training-only SMOTE, and threshold tuning to maximize the F2-score under a recall constraint of ≥0.70. Six classifiers were evaluated using average precision (AP), ROC-AUC, recall, precision, specificity, accuracy, and Brier score.

**Results:**

The major-amputation group exhibited a more severe baseline phenotype, including higher inflammatory burden, worse neuropathy and wound severity, and a higher prevalence of necrotizing fasciitis. Internally, multilayer perceptron achieved the highest AP (55.9% ± 13.6%). In external evaluation, k-nearest neighbors achieved the highest AP (65.1% ± 10.2%) and recall (72.8% ± 19.6%), whereas multilayer perceptron showed higher precision and specificity. Key contributors included necrotizing fasciitis, neuropathy severity, hemoglobin, PEDIS classification, and inflammatory indices.

**Conclusion:**

These findings suggest that prediction of amputation level is feasible, validated in a temporally separated cohort, and clinically interpretable, and may support future decision-support applications, although further validation is required before clinical implementation.

## 1 Introduction

Diabetes Mellitus (DM) is a widespread disease with rising global incidence and significant chronic complications. The increasing prevalence of DM has been closely associated with a higher burden of complications, including diabetic foot (DF) ulcers, which affect about 15–25% of patients throughout their lifetime (1). DF typically develops due to lower extremity neuropathy and/or peripheral arterial disease and is considered one of the most severe, costly, and debilitating complications of diabetes (2). DF ulcers significantly contribute to morbidity, accounting for over two-thirds of non-traumatic amputations and approximately 25% of hospitalizations among individuals with diabetes (3, 4). Globally, DF complications are the leading cause of lower limb amputation and mortality in hospitalized diabetic patients (5, 6).

Approximately 50% of patients with DF ulcers present with infections that often extend to the lower extremity’s deep fascial tissues and bones. Based on DF classification systems, infections classified in grades 3 and 4 typically demonstrate a progressive course that necessitates urgent surgical intervention (7). Early and aggressive surgical debridement and limited bone resection remain the cornerstone of DF surgical management.

The main goal of definitive DF surgery is to preserve the limb by maintaining the heel and ensuring a sufficient foot surface area. This approach aims to create a functional foot and improve survival outcomes. Major amputations are associated with a higher risk of mortality, highlighting the importance of limb preservation (5, 6). Pre-venting above-ankle amputation is essential for safeguarding cardiovascular health, maintaining metabolic balance, and enhancing overall quality of life.

A below-knee amputation, which is a major procedure, significantly increases mortality in patients with DF complications. The increased mortality is not attributed to the technical risks of surgery but rather to the loss of foot function caused by proximal amputation, which exacerbates metabolic derangements and associated mortality (8, 9). Patients undergoing major amputations exhibit poor rates of regaining mobility. Research indicates that 50-60% of patients with below-knee amputation regain mobility with the use of prosthetics, even among younger individuals. Additionally, the risk of mortality within six months following a major amputation is around 20%, highlighting the seriousness of this outcome. Mortality rates following major amputation in DF patients are comparable to those observed in malignancies (10, 11).

In DF with ischemia, infection, or osteomyelitis, amputations such as Chopart and Lisfranc-level procedures are commonly performed, often involving the preparation of flaps. However, postoperative complications, particularly surgical site infections and flap necrosis, are frequently encountered in this patient population (12, 13). The rates of complications and reamputation following these procedures in diabetic patients are notably higher compared to those undergoing isolated traumatic or oncological surgeries and are significantly elevated when compared to nondiabetic individuals (14). In cases of flap necrosis, the risk of reamputation and escalation to higher-level amputations is substantial. Reamputation rates of 28–43% have been reported following fore-foot and midfoot amputations (15, 16).

Transmetatarsal, Chopart, and Lisfranc amputations, which require flap surgery due to their technical complexity, are associated with high reamputation rates in DF patients. These rates vary depending on the technique employed (17, 18). The guillotine amputation technique, often utilized for emergency surgical intervention in cases of severe infection, is effective for rapid infection control and provides a larger surface area, thereby reducing the risk of flap necrosis. However, this approach may necessitate secondary interventions, such as wound closure or reconstruction with grafts, in the long term.

Accordingly, there is a clear need for a preoperative tool that can estimate whether an initially limb-preserving strategy is likely to culminate in minor or major amputation, thereby helping surgeons reduce the risk of reamputation and identify the most distal feasible level of surgery (9, 10, 12, 18, 19). Recent artificial intelligence applications in diabetic foot have shown that machine-learning models can support amputation-related prediction (20–23), while imaging-based AI models can aid diagnostic differentiation between osteomyelitis and Charcot neuroarthropathy (24, 25). Building on this rationale, the present study developed and temporally validated tested a machine-learning pipeline based on routinely available baseline clinical, laboratory, and selected imaging/vascular variables to predict minor versus major amputation after an initial limb-preserving approach. To facilitate real-world translation, the workflow was also designed for integration into a prototype web-based classification interface as part of a Software Architecture for Clinical Deployment. In addition, we aimed to characterize the underlying data structure through exploratory analyses, assess the stability of feature selection across repeated splits, compare classifier performance under internal and external evaluation, and interpret model behavior using explainable artificial intelligence.

## 2 Methods

### 2.1 Study design and ethical approval

Ethical approval for this retrospective study was obtained from the Health Sciences University, Gülhane Scientific Research Ethics Committee (Approval No: 2024/07; Decision No: 2024-443). All procedures were conducted in accordance with the Declaration of Helsinki and relevant institutional guidelines and regulations. Data access and usage were approved by the institutional data governance committee.

### 2.2 Patient population and definitions

Diabetic foot was defined based on the presence of infection, ulceration, or destruction of deep tissues associated with neuropathy and/or peripheral arterial disease in patients with diabetes. Inclusion criteria were: (i) age ≥18 years, (ii) diagnosis of Type 2 Diabetes Mellitus, (iii) clinical diagnosis of diabetic foot requiring surgical intervention, and (iv) undergoing an initial limb-preserving surgical approach. Exclusion criteria included: (i) non-diabetic foot etiologies, (ii) traumatic amputations, (iii) malignancy-related amputations, (iv) incomplete clinical or laboratory data, and (v) patients undergoing primary major amputation without an initial limb-preserving attempt.

Infection severity was classified using established systems such as PEDIS and Wagner classifications. Osteomyelitis was diagnosed based on a combination of clinical findings, imaging modalities (MRI or scintigraphy), and, where available, microbiological or histopathological confirmation.

### 2.3 Data structure and observation levels

In this study, the unit of analysis was the patient level. We conducted a retrospective study of diabetic patients who were clinically diagnosed with DF and underwent amputation between January 2016 and April 2024. A total of 628 unique patients were included. Each patient contributed a single preoperative sample corresponding to the presentation preceding the first limb-preserving surgical intervention, with all predictor variables recorded within a standardized preoperative time window. To prevent data leakage, no information from subsequent interventions or outcomes was included in feature construction.

### 2.4 Dataset construction and validation strategy

Dataset 1 and Dataset 2 were constructed as temporally separated cohorts for model development and temporal validation, respectively. Dataset 1 was derived from the primary institutional cohort and used for model training, internal validation, and hyperparameter optimization. Dataset 2 was reserved for temporal validation and was not involved in hyperparameter tuning, feature selection, or threshold optimization. This separation ensured an unbiased evaluation of model generalizability. Dataset separation was performed using a temporal split, where earlier cases were assigned to Dataset 1 and later cases to Dataset 2 to better simulate real-world model deployment. No patient contributed data to both Dataset 1 and Dataset 2. Although both datasets originated from the same overarching clinical population, this patient-level separation was applied to minimize overlap between cohorts.

### 2.5 Outcome measures and data collection

The primary outcome variable was defined as the level of amputation. Minor amputation (Class 0) included amputations distal to the ankle joint (toe, ray, transmetatarsal, Lisfranc, or Chopart levels), while major amputation (Class 1) was defined as any amputation proximal to the ankle, including below-knee amputations. Failure of the initial limb-preserving strategy was defined as progression to major amputation following an initial attempt at limb preservation.

All predictor variables were collected prior to the first limb-preserving surgical intervention, within a standardized 24–72 hour preoperative window. This ensured that the model reflects real-world preoperative decision-making conditions. Missing data were handled using median imputation within the machine-learning pipeline, and variables with excessive missingness were excluded prior to analysis.

For clarity, several clinically relevant variables are defined here. Neuropathy Severity was recorded according to the routine clinical assessment documented at admission and categorized based on the degree of peripheral neuropathy. CT utilization referred to whether computed tomography was performed during the initial preoperative evaluation. Cilostazol/Pentoxifylline use indicated active treatment with either medication at the time of admission. The number of arteries with monophasic flow represented the total number of lower-extremity arteries demonstrating monophasic Doppler waveforms on preoperative vascular ultrasonography, reflecting the extent of macrovascular arterial disease.

### 2.6 Exploratory data analysis and dataset characterization

Exploratory data analysis (EDA) was conducted on two temporally separated cohorts. In Dataset 1, there were n=538 observations in Class 0 (minor amputation) and n=55 in Class 1 (major amputation). Group-wise summaries and univariable comparisons for continuous/ordinal variables are provided in **Supplementary Material 1**, **Supplementary Table 1**, and the n(%) distributions and statistical comparisons for categorical variables are reported in **Supplementary Material 1**, **Supplementary Table 2**. In Dataset 2, the sample size was smaller (Class 0: n=26, Class 1: n=9), with corresponding continuous/ordinal and categorical summaries presented in **Supplementary Material 1**, **Supplementary Tables 3** and **S4**, respectively. A coding dictionary was used to standardize categorical/ordinal encodings and the clinical meaning of coded levels (**Supplementary Material 1**, **Supplementary Table 5**).

In total, 66 features were analyzed (39 continuous/ordinal and 27 categorical), spanning demographic/anthropometric, laboratory/metabolic, derived inflammatory, LRINEC-related, and clinical/imaging/vascular domains. For continuous variables, variance heterogeneity was assessed with Levene’s test. Welch’s t-test was used when appropriate and the Mann–Whitney U test otherwise, while ordinal variables were compared using the Mann–Whitney U test. Categorical variables were evaluated using Pearson’s chi-square test, with Fisher’s exact test applied to 2×2 tables when expected cell counts were small. For each feature, a primary p-value was defined based on the selected test and adjusted for multiple comparisons using both Benjamini–Hochberg FDR and Holm–Bonferroni procedures. FDR < 0.01 was prespecified to denote strong evidence after correction (**Supplementary Material 1**, **Supplementary Table 1**-**S4**). Continuous/ordinal variables are summarized with both mean ± SD and median (IQR) to reflect potential skewness and outlier sensitivity, which is particularly relevant for right-skewed inflammatory markers such as CRP, NLR, and PLR. Effect sizes were reported to complement significance testing (Cohen’s d for continuous variables and Cliff’s delta for ordinal variables; **Supplementary Material 1**, **Supplementary Tables 1**, **3**).

### 2.7 Machine-learning pipeline and evaluation workflow

**Figure 1:**
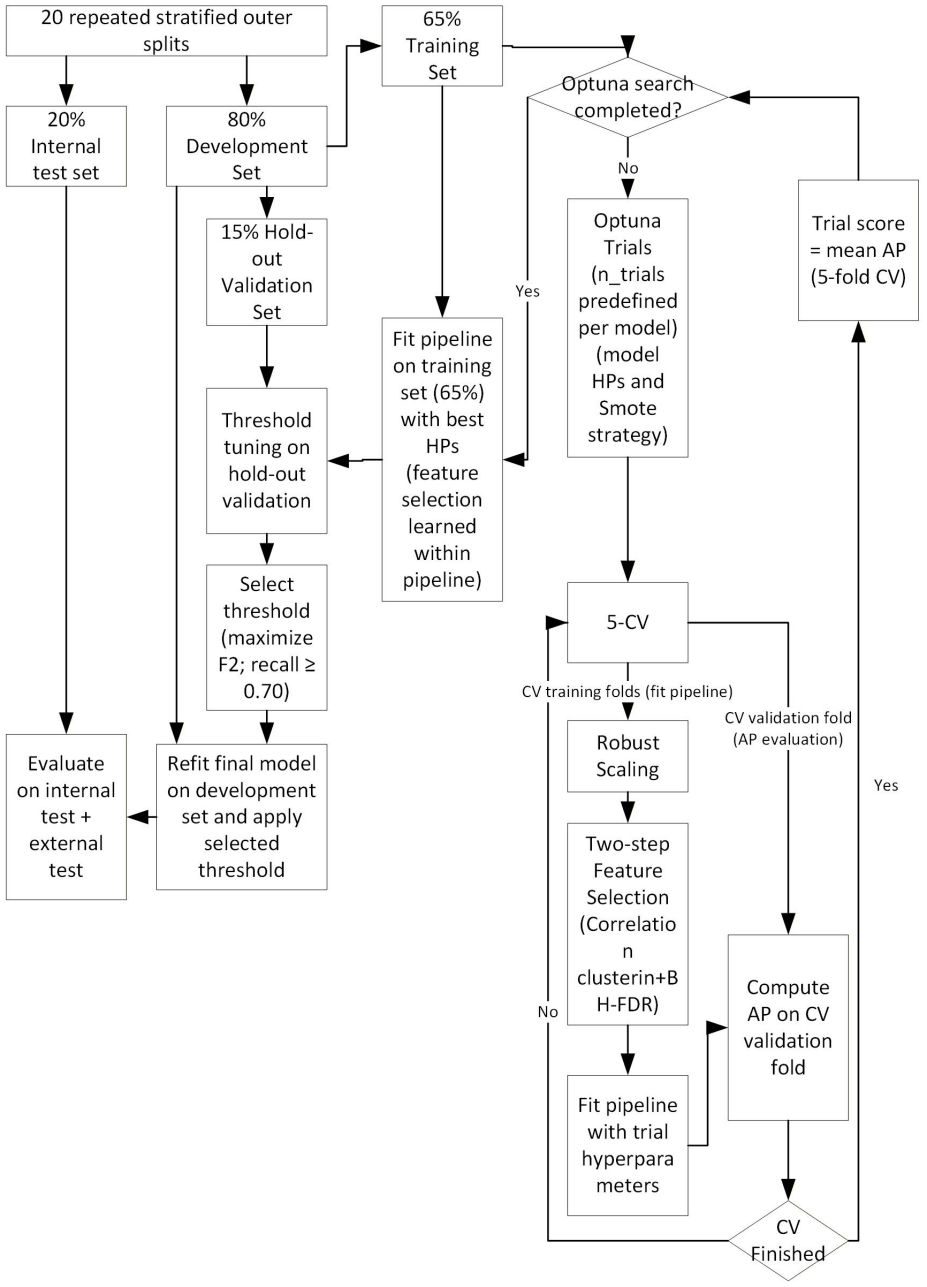
Overview of the nested model-development and evaluation workflow: 20 repeated stratified outer splits with Optuna-based hyperparameter/SMOTE optimization (5-fold CV on the 65% training set), hold-out validation for threshold tuning (maximize F2 with recall ≥ 0.70), and final evaluation on internal and external test sets.

A machine-learning classification pipeline was developed to distinguish minor amputation (Class 0) from major amputation (Class 1) using routinely collected clinical, laboratory, and selected imaging/vascular variables. Two temporally separated cohorts were used: Dataset 1 served as the development cohort and source of the internal test splits, while Dataset 2 was reserved exclusively for external testing. All records were labeled as Class 0 (minor amputation) or Class 1 (major amputation). Dataset 2 was not used for hyperparameter tuning, feature selection, or threshold optimization; however, external-cohort results were examined *post hoc* to select a representative repetition for interpretability and prototype demonstration. The full workflow is summarized in **Figure 1**.

Model development followed a 20-repetition stratified outer-split protocol. In each repetition, Dataset 1 was split into an internal test set (20%) and a development set (80%) using stratified sampling. The development set was then split into a training set (65% of the full Dataset 1) and a hold-out validation set (15% of the full Dataset 1), again using stratified sampling. This yields the same 65% training/15% hold-out validation/20% internal test partitioning. Hyperparameter search was performed only on the training set, threshold tuning was performed only on the hold-out validation set, and performance was reported on the internal test split and on the full temporal validation dataset 2.

All preprocessing steps were implemented within scikit-learn pipelines to prevent data leakage. Missing values were imputed using the median, and predictors were scaled using a robust scaling approach. These preprocessing steps were fit using training data only (within each cross-validation fold and within each repetition) and then applied to the corresponding validation/test data.

Feature selection was embedded in the pipeline and not tuned by Optuna. It consisted of two fixed steps applied after preprocessing. First, redundant predictors were reduced via correlation clustering: features with absolute pairwise correlation greater than 0.95 were grouped into clusters, and a single representative feature was selected from each cluster based on the strongest univariate association with the outcome in the training data (i.e., the feature showing the most discriminative univariate signal for Class 0 vs Class 1 within that training set). Second, these representative features were filtered using Benjamini–Hochberg false discovery rate (FDR) control at q = 0.10, retaining only those that passed the FDR criterion; to avoid degenerate cases, at least one feature was retained by construction when necessary. This corresponds to the “two-step feature selection (correlation clustering + BH-FDR)” block in **Figure 1**.

To address class imbalance, SMOTE oversampling was applied only to the training data within the pipeline, after preprocessing and feature selection. During hyperparameter optimization, both the target degree of oversampling (how strongly the minority class is synthesized) and the number of nearest neighbors used to generate synthetic samples were tuned jointly with model hyperparameters. No oversampling was applied to validation or test data.

Six classifiers were evaluated: Random Forest, Support Vector Machine (SVM), Multilayer Perceptron (MLP), XGBoost, k-Nearest Neighbors (KNN), and Bagging. Hyperparameter optimization was carried out with Optuna using a TPE sampler. For each model in each repetition, Optuna evaluated candidate configurations using 5-fold StratifiedKFold cross-validation on the training set, and the optimization objective was the mean Average Precision (AP) across folds. The number of Optuna trials was fixed per model (80 trials for Random Forest, SVM, MLP, and XGBoost; 60 trials for KNN and Bagging). The hyperparameter search space for each model and the SMOTE-related tuning ranges are reported in **Table 1**.

**Table 1:** Optuna hyperparameter search space for SMOTE and all evaluated classifiers.

Global (applied to all models; SMOTE)			XGBoost		
Hyperparameter	Search space	Type / distribution	Hyperparameter	Search space	Type / distribution
sampling_strategy	{0.3, 0.5, 0.7, 1.0}	categorical	n_estimators	200–800 (step 100)	integer
k_neighbors	3–7	integer	max_depth	2–10	integer
Random forest			learning_rate	1e−3–3e−1	log-uniform
Hyperparameter	Search space	Type / distribution	subsample	0.5–1.0	uniform
n_estimators	200–800 (step 100)	integer	colsample_bytree	0.5–1.0	uniform
max_depth	2–30	integer	min_child_weight	1e−1–10	log-uniform
min_samples_split	2–20	integer	gamma	0–5	uniform
min_samples_leaf	1–10	integer	reg_alpha	1e−8–1.0	log-uniform
max_features	{“sqrt”, “log2”, None}	categorical	reg_lambda	1e−3–10	log-uniform
criterion	{“gini”, “entropy”, “log_loss”}	categorical	scale_pos_weight*	0.5×r – 2.0×r	log-uniform
bootstrap	{True, False}	categorical	* r = (number of negatives / number of positives) computed on the training set used in that repetition.		
Support vector machine (SVM)			k-Nearest neighbors (KNN)		
Hyperparameter	Search space	Type / distribution	Hyperparameter	Search space	Type / distribution
kernel	{“linear”, “rbf”, “poly”}	categorical	n_neighbors	1–30	integer
C	1e−3–1e3	log-uniform	weights	{“uniform”, “distance”}	categorical
gamma_mode*	{“scale”, “auto”, “float”}	categorical	p	1–2	integer
gamma**	1e−4–1.0	log-uniform	leaf_size	10–60	integer
degree***	2–5	integer	metric	{“minkowski”, “euclidean”, “manhattan”}	categorical
* sampled only if kernel ∈ {rbf, poly}			Bagging		
** sampled only if gamma_mode = “float”			Hyperparameter	Search space	Type / distribution
*** sampled only if kernel = “poly”			base_estimator	{“tree”, “knn”}	categorical
Multilayer perceptron (MLP)			n_estimators	10–200 (step 10)	integer
Hyperparameter	Search space	Type / distribution	max_samples	0.5–1.0	uniform
solver	{“adam”, “lbfgs”}	categorical	max_features	0.5–1.0	uniform
hidden_layer_sizes	{(20,10,5), (15,15,5), (20,15,5), (20,10)}	categorical	bootstrap	{True, False}	categorical
activation	{“relu”, “tanh”, “logistic”}	categorical	bootstrap_features	{False, True}	categorical
alpha	1e−5–1e−1	log-uniform	Base estimator if “tree”		
max_iter	400–1200 (step 100)	integer	Hyperparameter	Search space	Type
learning_rate*	{“constant”, “adaptive”}	categorical	max_depth	1–15	integer
learning_rate_init*	1e−5–1e−3	log-uniform	min_samples_split	2–10	integer
beta_1*	0.70–0.99	uniform	min_samples_leaf	1–5	integer
beta_2*	0.90–0.999	uniform	Base estimator if “knn”		
early_stopping*	{True, False}	categorical	Hyperparameter	Search space	Type
batch_size*	{16, 32, 64}	categorical	n_neighbors	3–15	integer
* sampled only if solver = “adam”			weights	{“uniform”, “distance”}	categorical

For each model, hyperparameters were optimized by maximizing mean average precision (AP) across 5-fold StratifiedKFold cross-validation on the training set.

After Optuna identified the best hyperparameters for a given model in a given repetition, the pipeline was fit on the training set (65%) and a decision threshold was tuned on the hold-out validation set (15%). Threshold selection was performed by scanning 200 candidate thresholds between 0.01 and 0.99 and choosing the threshold that maximized the F2-score under a minimum recall constraint of 0.70. If no threshold satisfied the recall constraint, a default threshold of 0.50 was used. The final model was then refit on the full development set (80%) using the selected hyperparameters, and the selected threshold was applied without further modification. Performance was evaluated on the internal test split of Dataset 1 (20%) and the temporal validation dataset 2, and results were aggregated across the 20 repetitions.

Model performance was reported using both threshold-free and threshold-dependent metrics. Threshold-free metrics included ROC-AUC and Average Precision (AP). Threshold-dependent metrics included F1-score, recall, and F2-score (using the selected operating threshold), along with confusion-matrix–derived measures such as precision, specificity, and accuracy. Probability calibration was assessed using the Brier score and fixed-bin calibration curves (10 uniform bins). ROC, precision–recall, and calibration curve points were stored for each repetition and summarized as mean ± standard deviation for internal (Dataset 1) and external (Dataset 2) evaluations.

For reproducibility, the training process automatically saved fitted models (joblib), per-model and per-repetition metadata (JSON), a consolidated model registry (registry JSON), raw results and summary performance tables (CSV), curve-point objects (joblib), and mean ± SD plots (PNG). For XGBoost, the fitted classifier was additionally exported in its native JSON format. These stored artifacts were retained not only for auditability and result reproduction, but also to enable deployment of the trained models and the corresponding selected-feature pipelines within a prototype web-based classification interface for minor–major amputation classification. For transparency and reproducibility, the project repository associated with this study is publicly available at https://github.com/DrGokalpTulum/diabeticfootai.

### 2.8 Prototype web-based decision-support interface

To support translation of the trained prediction framework into a clinician-facing environment, we developed a prototype web-based decision-support interface for minor-versus-major amputation classification. The prototype operationalized the stored trained models and their corresponding selected-feature inference pipelines generated during model development. For deployment, a fixed 23-feature input schema was used, corresponding to the common feature subset retained in repetition 11; the deployed repetition 11 pipelines, spreadsheet template, and manual-entry workflow were built around this shared subset. The interface accepts either single-patient spreadsheet upload or structured manual entry through a three-step form covering laboratory/vital variables, clinical findings, and summary scores. Within a single session, the interface presents the outputs of six classifiers side-by-side (Bagging, KNN, MLP, Random Forest, SVM, and XGBoost). After inference, the system displays the predicted class and associated confidence for each of the six deployed models, together with a feature-distribution plot that places the new patient against the historical cohort using user-selected feature pairs from the deployment subset. A validation modal allows the clinician to label the system output as correct, incorrect, or unsure, thereby supporting later auditing and future model evaluation. The prototype was designed as a research-oriented decision-support aid and should not be interpreted as a replacement for multidisciplinary clinical judgment. Additional implementation and workflow details are provided in the **Supplementary Material 2**. A public-facing deployment associated with this study is hosted at https://diabeticfootai.com/.

## 3 Results

### 3.1 Exploratory data analysis findings

In Dataset 1, the major amputation group exhibited a clear shift toward a higher inflammatory/infectious burden, together with higher clinical severity. WBC, NEU, and CRP were higher in Class 1 (WBC: Class 0: 10.67 ± 4.80; Class 1: 15.15 ± 6.65; NEU: Class 0: 7.73 ± 4.39; Class 1: 12.56 ± 6.33; CRP: Class 0: 72.68 ± 82.02; Class 1: 139.94 ± 88.13). In contrast, Hb and Serum Albumin were lower (Hb: Class 0: 11.53 ± 4.63; Class 1: 9.72 ± 1.46; Serum Albumin: Class 0: 3.56 ± 1.81; Class 1: 3.06 ± 0.51). Derived indices also increased in Class 1 (NLR: Class 0: 4.99 ± 4.30; Class 1: 12.73 ± 11.99; PLR: Class 0: 208.23 ± 112.96; Class 1: 322.81 ± 211.45) (**Supplementary Material 1**, **Supplementary Table 1**).

Along the LRINEC axis, the LRINEC Score was higher in the major amputation group (Class 0: 3.41 ± 2.68; Class 1: 6.11 ± 3.41). Clinical severity indicators were also higher, including ASA Physical Status Classification (Class 0: 2.86 ± 0.47; Class 1: 3.11 ± 0.42), Wagner Classification (Class 0: 3.83 ± 0.38; Class 1: 4.02 ± 0.30), PEDIS Classification (Class 0: 2.46 ± 0.50; Class 1: 2.89 ± 0.37), and Neuropathy Severity (Class 0: 1.02 ± 1.16; Class 1: 1.89 ± 1.05). Several of these differences remained significant after multiple-comparison correction at FDR < 0.01, and the corresponding effect sizes were consistent with meaningful separation between groups (**Supplementary Material 1**, **Supplementary Table 1**).

Among categorical features in Dataset 1, the most prominent between-group differences involved Ambulation Status, Cilostazol/Pentoxifylline use, Computed Tomography (CT) use, and Necrotizing Fasciitis. All of these remained significant after correction at FDR < 0.01 (**Supplementary Material 1**, **Supplementary Table 2**). These findings were accompanied by lower ambulation, more frequent Cilostazol/Pentoxifylline use, more frequent CT use, and a higher prevalence of Necrotizing Fasciitis in the major amputation group.

In Dataset 2, fewer between-group differences remained significant after multiple-testing correction (**Supplementary Material 1**, **Supplementary Tables 3**, **4**). Nevertheless, directional trends broadly mirrored Dataset 1 across several inflammation and infection-related measures. In the categorical analysis, CT use showed evidence of a between-group difference before correction (primary p-value < 0.01). However, this association did not remain significant after Benjamini–Hochberg FDR adjustment (FDR-adjusted p = 0.09) (**Supplementary Material 1**, **Supplementary Table 4**).

### 3.2 Feature selection results

**Figure 2:**
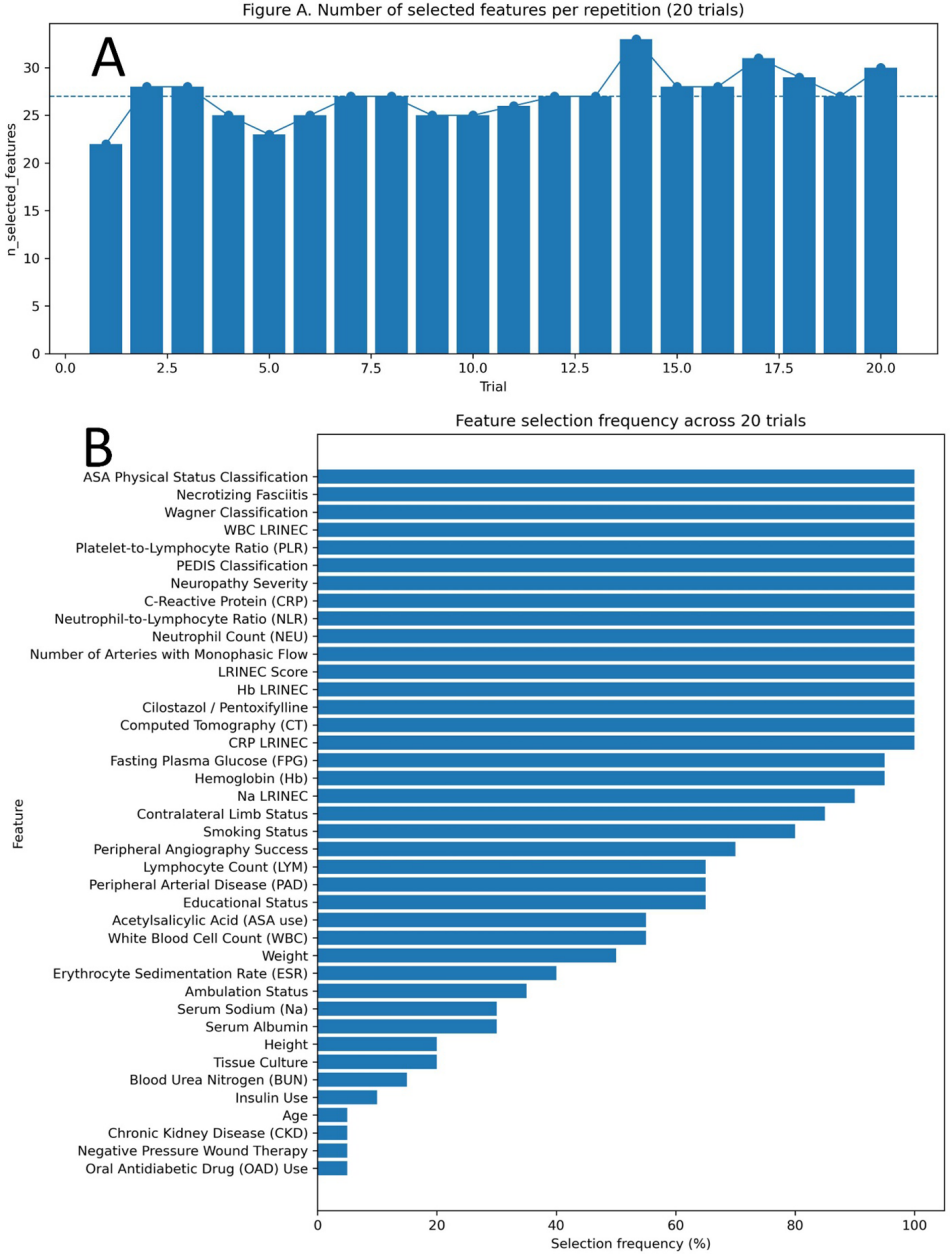
Stability of the two-step feature selection procedure across 20 repetitions. **(A)** Number of selected features per repetition. **(B)** Selection frequency of individual features across repetitions.

Across the 20 repeated stratified outer splits, 22–33 features were selected per repetition by the embedded feature selection procedure (**Figure 2A**). The selection-frequency analysis in **Figure 2B** identified a stable core set of variables retained consistently across repetitions. Features selected in 100% of repetitions were ASA Physical Status Classification, C-reactive protein (CRP), CRP-LRINEC, Computed Tomography (CT), Cilostazol/Pentoxifylline use, Hb-LRINEC, LRINEC score, Number of arteries with monophasic flow, Neutrophil count (NEU), Necrotizing Fasciitis, Neutrophil-to-lymphocyte ratio (NLR), Neuropathy Severity, PEDIS Classification, Platelet-to-lymphocyte ratio (PLR), WBC-LRINEC, and Wagner classification. In addition, variables such as fasting plasma glucose (FPG) and hemoglobin (Hb) were retained in the large majority of repetitions, whereas a smaller subset of features appeared only intermittently across runs.

### 3.3 Model performance on internal testing and temporal validation

**Figure 3:**
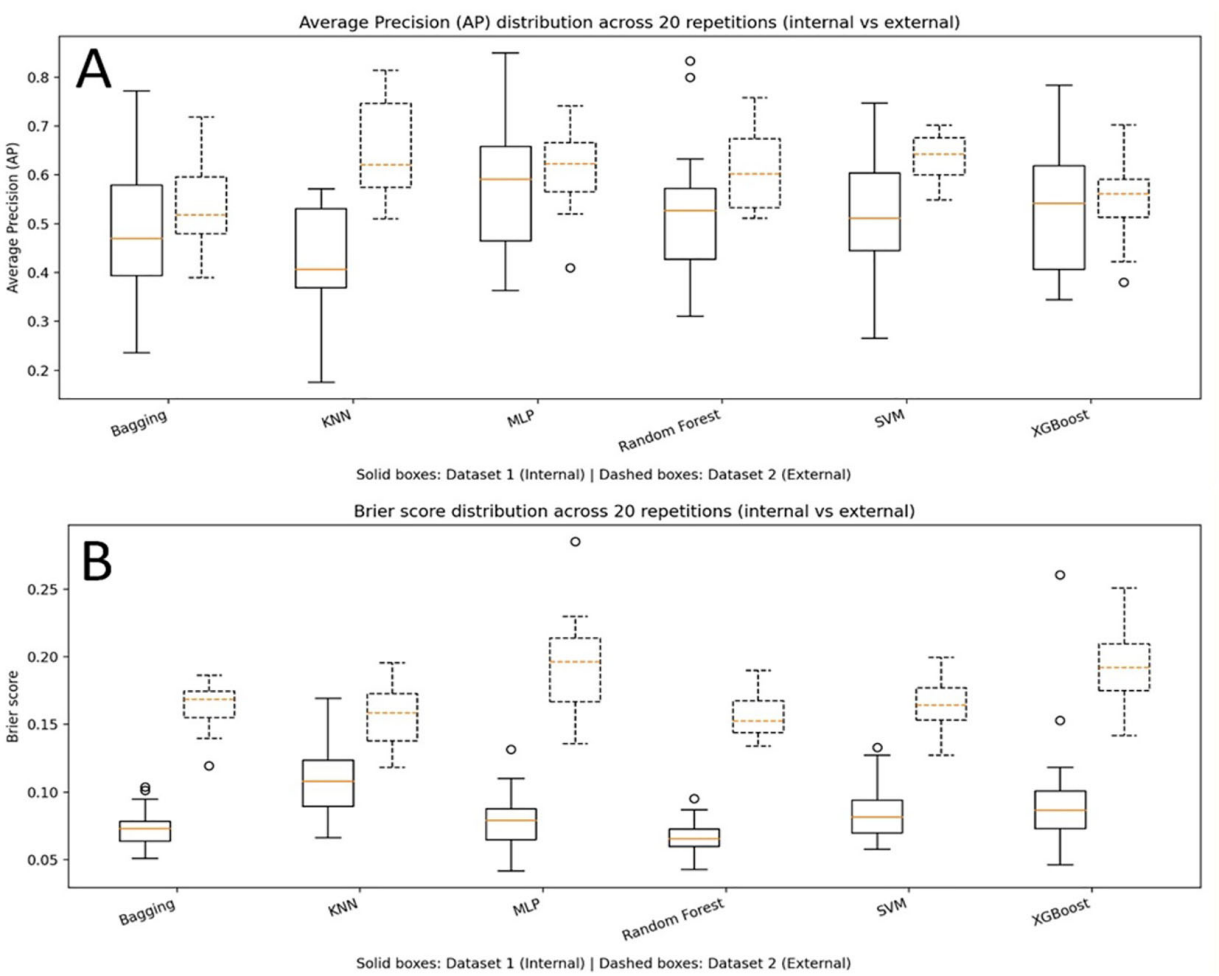
Distribution of model performance across 20 repetitions. **(A)** Boxplots of Average Precision (AP) for the internal and external test sets. **(B)** Boxplots of Brier scores for the internal and external test sets.

Overall model performance across the 20 repeated stratified outer splits is summarized in **Table 2**, while the distributions of Average Precision (AP) and Brier score across repetitions are shown in **Figure 3**.

**Table 2:** Summary of performance metrics of the evaluated classifiers on the internal and external test sets across 20 repetitions.

Internal cohort										
Model	Threshold	AUC	AP	F1	Recall	F2	Precision	Specificity	Accuracy	Brier
Bagging	0.27 ± 0.12 (95% CI: 0.22–0.32)	87.2% ± 5.8% (95% CI: 84.7%–89.7%)	48.5% ± 14.2% (95% CI: 42.3%–54.7%)	44.5% ± 10.5% (95% CI: 39.9%–49.1%)	70.5% ± 17.2% (95% CI: 62.9%–78.0%)	55.9% ± 11.5% (95% CI: 50.9%–60.9%)	34.6% ± 11.4% (95% CI: 29.7%–39.6%)	83.5% ± 11.0% (95% CI: 78.7%–88.3%)	82.3% ± 9.3% (95% CI: 78.2%–86.4%)	0.073 ± 0.015 (95% CI: 0.066–0.079)
KNN	0.42 ± 0.23 (95% CI: 0.32–0.52)	85.8% ± 4.8% (95% CI: 83.7%–88.0%)	42.6% ± 10.8% (95% CI: 37.9%–47.3%)	38.9% ± 9.1% (95% CI: 34.9%–42.9%)	70.9% ± 25.0% (95% CI: 60.0%–81.8%)	52.1% ± 14.4% (95% CI: 45.8%–58.4%)	29.8% ± 8.2% (95% CI: 26.2%–33.4%)	80.6% ± 9.9% (95% CI: 76.3%–85.0%)	79.7% ± 7.2% (95% CI: 76.6%–82.9%)	0.110 ± 0.028 (95% CI: 0.098–0.122)
MLP	0.29 ± 0.22 (95% CI: 0.19–0.39)	88.5% ± 5.8% (95% CI: 85.9%–91.0%)	55.9% ± 13.6% (95% CI: 49.9%–61.8%)	52.7% ± 9.3% (95% CI: 48.6%–56.8%)	70.0% ± 17.2% (95% CI: 62.5%–77.5%)	61.3% ± 12.5% (95% CI: 55.8%–66.8%)	45.2% ± 12.3% (95% CI: 39.8%–50.6%)	90.2% ± 4.4% (95% CI: 88.2%–92.1%)	88.3% ± 3.6% (95% CI: 86.7%–89.9%)	0.080 ± 0.021 (95% CI: 0.070–0.089)
Random Forest	0.22 ± 0.10 (95% CI: 0.17–0.26)	88.8% ± 4.8% (95% CI: 86.7%–90.9%)	51.8% ± 13.9% (95% CI: 45.7%–57.9%)	45.1% ± 14.2% (95% CI: 38.9%–51.4%)	75.9% ± 17.5% (95% CI: 68.2%–83.6%)	58.0% ± 13.6% (95% CI: 52.1%–64.0%)	34.8% ± 16.0% (95% CI: 27.8%–41.9%)	81.0% ± 12.9% (95% CI: 75.4%–86.7%)	80.5% ± 11.1% (95% CI: 75.7%–85.4%)	0.067 ± 0.012 (95% CI: 0.062–0.072)
SVM	0.30 ± 0.22 (95% CI: 0.20–0.40)	86.8% ± 6.3% (95% CI: 84.1%–89.6%)	52.4% ± 13.2% (95% CI: 46.7%–58.2%)	45.0% ± 8.1% (95% CI: 41.5%–48.5%)	74.5% ± 20.6% (95% CI: 65.5%–83.6%)	57.6% ± 11.1% (95% CI: 52.8%–62.5%)	35.0% ± 11.2% (95% CI: 30.1%–39.9%)	83.1% ± 10.5% (95% CI: 78.5%–87.7%)	82.3% ± 8.4% (95% CI: 78.6%–86.0%)	0.085 ± 0.021 (95% CI: 0.076–0.094)
XGBoost	0.31 ± 0.25 (95% CI: 0.21–0.42)	88.9% ± 5.2% (95% CI: 86.6%–91.2%)	52.6% ± 13.0% (95% CI: 47.0%–58.3%)	44.4% ± 9.3% (95% CI: 40.3%–48.5%)	70.5% ± 18.4% (95% CI: 62.4%–78.5%)	56.0% ± 11.4% (95% CI: 51.0%–60.9%)	34.5% ± 9.9% (95% CI: 30.2%–38.8%)	84.3% ± 8.9% (95% CI: 80.4%–88.2%)	83.0% ± 7.2% (95% CI: 79.8%–86.1%)	0.095 ± 0.047 (95% CI: 0.074–0.115)
External cohort										
Model	Threshold	AUC	AP	F1	Recall	F2	Precision	Specificity	Accuracy	Brier
Bagging	0.27 ± 0.12 (95% CI: 0.22–0.32)	76.3% ± 4.8% (95% CI: 74.2%–78.4%)	54.0% ± 9.9% (95% CI: 49.6%–58.3%)	48.3% ± 11.4% (95% CI: 43.3%–53.3%)	55.0% ± 18.2% (95% CI: 47.0%–63.0%)	51.5% ± 13.9% (95% CI: 45.5%–57.6%)	46.6% ± 11.7% (95% CI: 41.5%–51.8%)	75.8% ± 13.8% (95% CI: 69.7%–81.8%)	70.4% ± 8.2% (95% CI: 66.8%–74.0%)	0.164 ± 0.017 (95% CI: 0.156–0.171)
KNN	0.42 ± 0.23 (95% CI: 0.32–0.52)	83.7% ± 3.4% (95% CI: 82.2%–85.2%)	65.1% ± 10.2% (95% CI: 60.6%–69.6%)	57.2% ± 9.2% (95% CI: 53.1%–61.2%)	72.8% ± 19.6% (95% CI: 64.2%–81.4%)	64.9% ± 13.9% (95% CI: 58.8%–71.0%)	51.3% ± 13.8% (95% CI: 45.2%–57.3%)	72.5% ± 13.7% (95% CI: 66.5%–78.5%)	72.6% ± 7.1% (95% CI: 69.4%–75.7%)	0.157 ± 0.022 (95% CI: 0.147–0.166)
MLP	0.29 ± 0.22 (95% CI: 0.19–0.39)	79.9% ± 4.4% (95% CI: 78.0%–81.8%)	61.5% ± 7.8% (95% CI: 58.0%–64.9%)	56.8% ± 9.2% (95% CI: 52.8%–60.8%)	59.4% ± 12.1% (95% CI: 54.1%–64.7%)	58.1% ± 10.5% (95% CI: 53.6%–62.7%)	56.2% ± 11.3% (95% CI: 51.3%–61.2%)	82.9% ± 7.2% (95% CI: 79.7%–86.0%)	76.9% ± 5.4% (95% CI: 74.5%–79.2%)	0.192 ± 0.036 (95% CI: 0.176–0.208)
Random Forest	0.22 ± 0.10 (95% CI: 0.17–0.26)	80.2% ± 3.3% (95% CI: 78.8%–81.7%)	61.3% ± 8.0% (95% CI: 57.8%–64.8%)	51.4% ± 12.8% (95% CI: 45.7%–57.0%)	63.9% ± 21.6% (95% CI: 54.4%–73.4%)	57.6% ± 16.8% (95% CI: 50.3%–65.0%)	46.2% ± 8.7% (95% CI: 42.3%–50.0%)	72.9% ± 14.1% (95% CI: 66.7%–79.1%)	70.6% ± 7.4% (95% CI: 67.3%–73.8%)	0.157 ± 0.016 (95% CI: 0.150–0.164)
SVM	0.30 ± 0.22 (95% CI: 0.20–0.40)	79.7% ± 3.1% (95% CI: 78.4%–81.1%)	63.8% ± 4.8% (95% CI: 61.7%–65.9%)	55.4% ± 6.4% (95% CI: 52.5%–58.2%)	65.0% ± 15.0% (95% CI: 58.4%–71.6%)	60.3% ± 10.1% (95% CI: 55.8%–64.7%)	50.9% ± 8.9% (95% CI: 47.0%–54.8%)	76.2% ± 11.7% (95% CI: 71.0%–81.3%)	73.3% ± 6.1% (95% CI: 70.6%–76.0%)	0.164 ± 0.020 (95% CI: 0.156–0.173)
XGBoost	0.31 ± 0.25 (95% CI: 0.21–0.42)	77.9% ± 4.9% (95% CI: 75.7%–80.0%)	54.9% ± 7.9% (95% CI: 51.5%–58.4%)	48.5% ± 12.3% (95% CI: 43.1%–53.9%)	56.7% ± 23.3% (95% CI: 46.4%–66.9%)	52.4% ± 17.5% (95% CI: 44.7%–60.1%)	46.6% ± 10.2% (95% CI: 42.2%–51.1%)	76.3% ± 12.8% (95% CI: 70.7%–82.0%)	71.3% ± 5.7% (95% CI: 68.8%–73.8%)	0.193 ± 0.027 (95% CI: 0.181–0.204)

In the internal test splits of Dataset 1, threshold-free discrimination was consistently strong, with mean ROC-AUC values ranging from 85.8% for KNN to 88.9% for XGBoost. In terms of AP, the highest internal mean value was achieved by MLP (55.9% ± 13.6%), followed by XGBoost (52.6% ± 13.0%), SVM (52.4% ± 13.2%), and Random Forest (51.8% ± 13.9%), whereas KNN yielded the lowest internal AP (42.6% ± 10.8%). Internal recall values remained within a relatively narrow band across models (70.0%–75.9%), while Random Forest achieved the lowest internal Brier score (0.067 ± 0.012).

In temporal validation on Dataset 2, ROC-AUC and calibration deteriorated overall, whereas Average Precision improved and the ranking of classifiers changed. The selected operating thresholds remained lower for Random Forest and higher for KNN across both datasets. Mean ROC-AUC values ranged from 76.3% to 83.7%, and the ranking by AP differed from that observed internally. KNN achieved the highest external AP (65.1% ± 10.2%), followed by SVM (63.8% ± 4.8%), MLP (61.5% ± 7.8%), and Random Forest (61.3% ± 8.0%), whereas Bagging (54.0% ± 9.9%) and XGBoost (54.9% ± 7.9%) showed lower external AP values. External recall separated more clearly across classifiers, with KNN maintaining the highest mean recall (72.8% ± 19.6%). In contrast, threshold-dependent metrics such as precision, specificity, and accuracy were highest for MLP, which achieved the best external precision (56.2% ± 11.3%), specificity (82.9% ± 7.2%), and accuracy (76.9% ± 5.4%). The lowest external Brier scores were observed for Random Forest (0.157 ± 0.016) and KNN (0.157 ± 0.022).

### 3.4 Internal cohort

**Figure 4:**
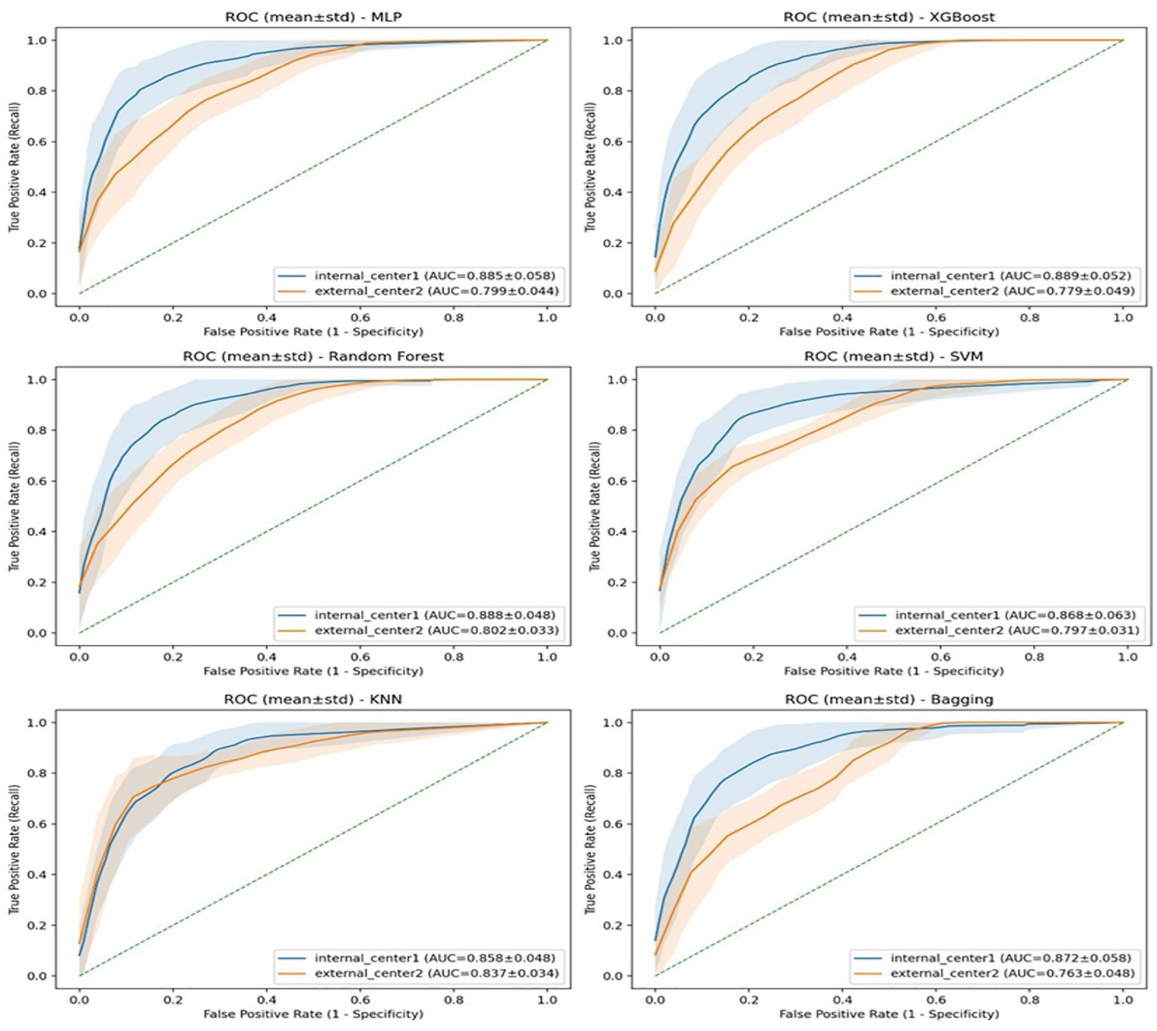
Mean ROC curves of the evaluated classifiers on the internal and external test sets across 20 repetitions.

The mean ROC curves for the internal and external evaluations are shown in **Figure 4**. In the internal cohort, the ROC curves were relatively closely clustered, consistent with the narrow range of AUC values reported in **Table 2**. In the external cohort, the separation between models became more visible. KNN maintained the most favorable ROC profile, while Bagging showed the weakest discrimination. The remaining models occupied an intermediate range with broadly similar external ROC behavior.

**Figure 5:**
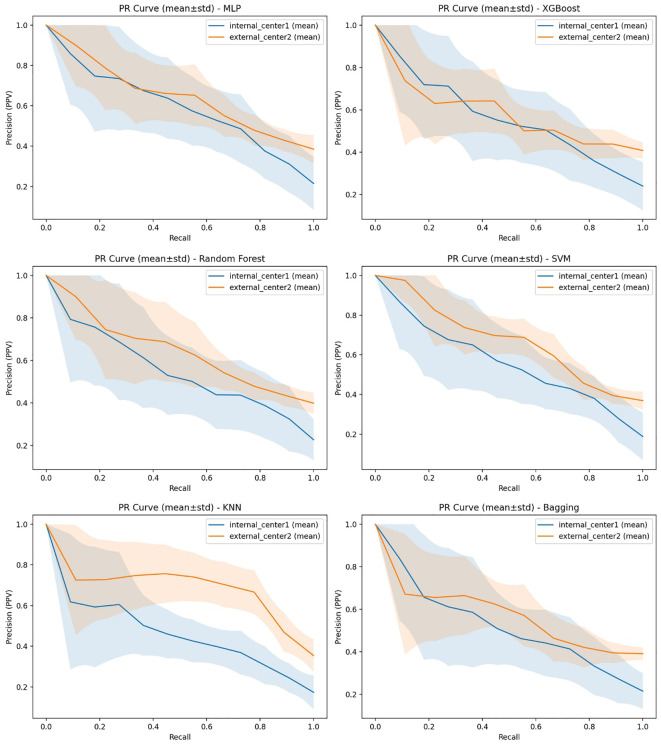
Mean Precision–Recall curves of the evaluated classifiers on the internal and external test sets across 20 repetitions.

Mean precision–recall curves are presented in **Figure 5**. In the internal cohort, MLP, SVM, Random Forest, and XGBoost followed broadly similar PR profiles, while KNN remained lower over much of the recall range. In the external cohort, this ordering changed. KNN preserved higher precision across a wider span of recall values and showed the strongest overall PR profile, whereas Bagging and XGBoost tended to occupy lower regions of the PR space. SVM, MLP, and Random Forest formed an intermediate group with relatively similar external behavior.

The AP distributions in **Figure 3A** are consistent with these PR patterns. Internally, KNN showed a lower median AP and a narrower upper tail than the other classifiers, whereas externally its AP distribution was shifted upward relative to the remaining models.

Paired comparisons of AP across the 20 repetitions were conducted using the Wilcoxon signed-rank test with Holm correction for multiple testing, and the results are summarized in **Table 3**. Internal and external paired AP comparisons are presented as separate blocks. On internal testing, KNN was significantly outperformed by MLP, Random Forest, SVM, and XGBoost after multiplicity adjustment. Bagging also performed significantly worse than MLP. By contrast, most pairwise differences among the leading internal performers (MLP, SVM, XGBoost, and Random Forest) were not statistically significant after Holm correction. On temporal validation, KNN achieved significantly higher AP than Bagging and XGBoost. In addition, Random Forest and SVM significantly outperformed both Bagging and XGBoost after Holm correction. No other pairwise differences remained significant.

**Table 3:** Pairwise comparisons of average precision (AP) between classifiers using the Wilcoxon signed-rank test with Holm correction.

		Internal cohort				External cohort				
Metric	Comparison	Δ Mean	Wilcoxon p	Holm-adj p	sig (Holm<0.05)	Δ Mean	Wilcoxon p	Holm-adj p	sig (Holm<0.05)	N paired reps
AP	KNN vs. Bagging	-5.89	0.044	0.308	ns	11.14	0.001	0.015	yes	20
	KNN vs. MLP	-13.27	<0.001	0.004	yes	3.65	0.43	1	ns	20
	KNN vs. Random Forest	-9.18	0.001	0.008	yes	3.78	0.097	0.681	ns	20
	KNN vs. SVM	-9.84	<0.001	0.005	yes	1.34	0.596	1	ns	20
	KNN vs. XGBoost	-10.03	0.001	0.015	yes	10.2	0.002	0.022	yes	20
	Bagging vs. MLP	-7.38	0.001	0.015	yes	-7.5	0.015	0.138	ns	20
	Bagging vs. Random Forest	-3.29	0.021	0.193	ns	-7.37	<0.001	0.006	yes	20
	Bagging vs. SVM	-3.95	0.033	0.262	ns	-9.81	<0.001	0.003	yes	20
	Bagging vs. XGBoost	-4.14	0.007	0.073	ns	-0.94	0.622	1	ns	20
	MLP vs. Random Forest	4.08	0.133	0.664	ns	0.13	1	1	ns	20
	MLP vs. SVM	3.43	0.105	0.632	ns	-2.31	0.475	1	ns	20
	MLP vs. XGBoost	3.24	0.165	0.664	ns	6.56	0.017	0.138	ns	20
	Random Forest vs. SVM	-0.66	0.622	1	ns	-2.44	0.154	0.922	ns	20
	Random Forest vs. XGBoost	-0.85	0.756	1	ns	6.42	0.004	0.042	yes	20
	SVM vs. XGBoost	-0.19	0.784	1	ns	8.87	<0.001	0.001	yes	20

**Figure 6:**
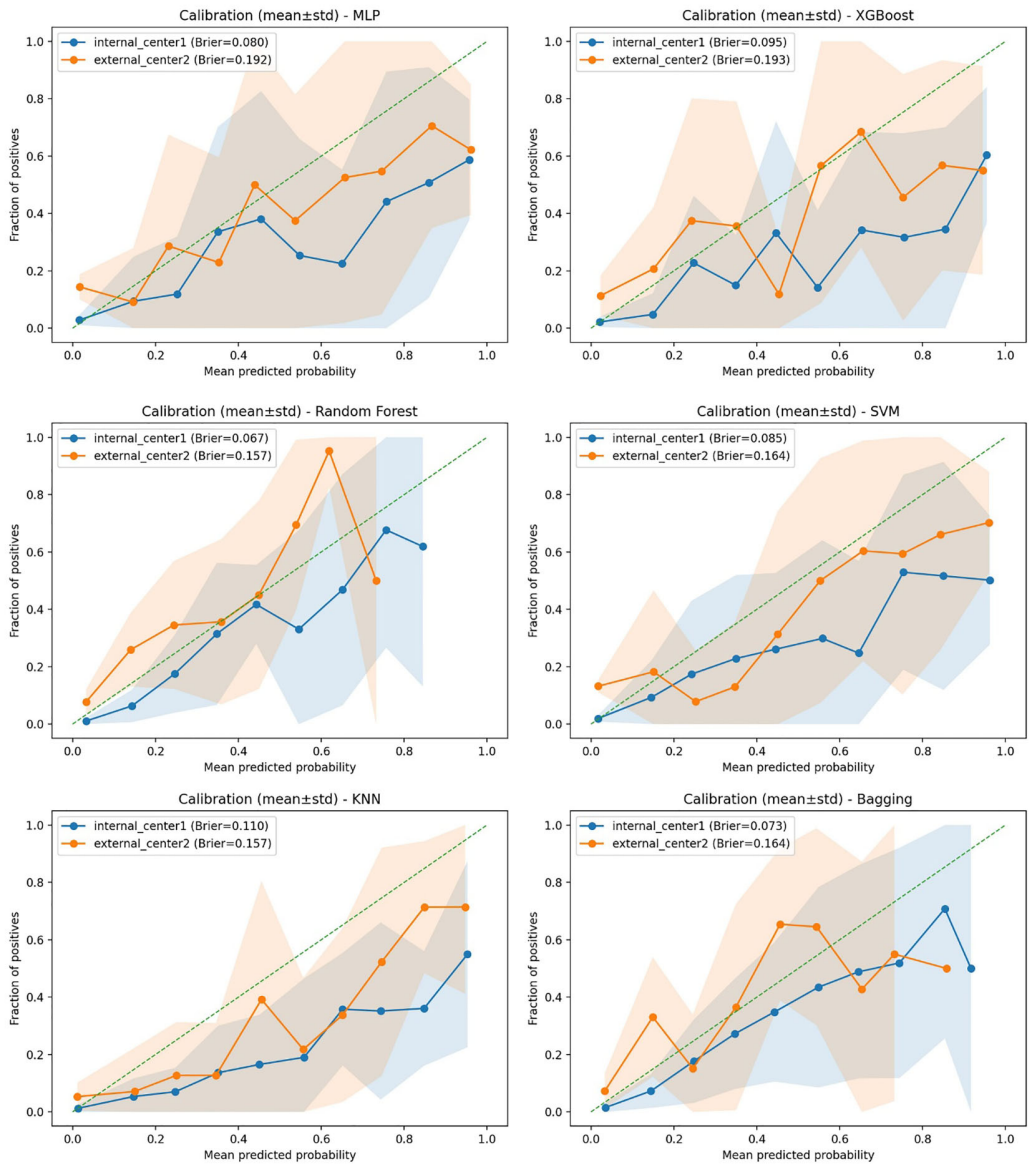
Mean calibration curves of the evaluated classifiers on the internal and external test sets across 20 repetitions.

Calibration was assessed using Brier scores and aggregated calibration curves, with the corresponding curves shown in **Figure 6** and the Brier score distributions shown in **Figure 3B**. The calibration plots indicate that deviations from the diagonal were generally more pronounced in Dataset 2 than in Dataset 1. In the internal cohort, several models followed the reference line reasonably closely across much of the probability range. In the external cohort, calibration deteriorated overall, with larger departures from the ideal line.

Among the evaluated classifiers, Random Forest and KNN remained closer to the diagonal than the other models over substantial portions of the curve. The Brier score distributions in **Figure 3B** support this pattern, showing an upward shift from internal testing to temporal validation across all models, with relatively lower external Brier distributions for Random Forest and KNN, and higher distributions for MLP and XGBoost.

For the XAI analyses, the Random Forest model from repetition 11 was selected as the primary interpretability model after considering performance across repeated experiments. This model was chosen not only because of its strong internal performance, but also because it maintained a balanced profile in the external cohort in terms of discrimination and calibration. To minimize split-related variability in the interpretability analysis, all main XAI figures were generated from this repetition-model combination. The prototype itself was implemented as a multi-model interface that supported side-by-side inference from all six deployed classifiers. In contrast, the Random Forest model from repetition 11 was used only as the primary interpretability model for the main XAI figures. Comparative XAI outputs for the MLP model from the same repetition are provided in **Supplementary Material 1**, **Supplementary Figure 1**.

**Figure 7:**
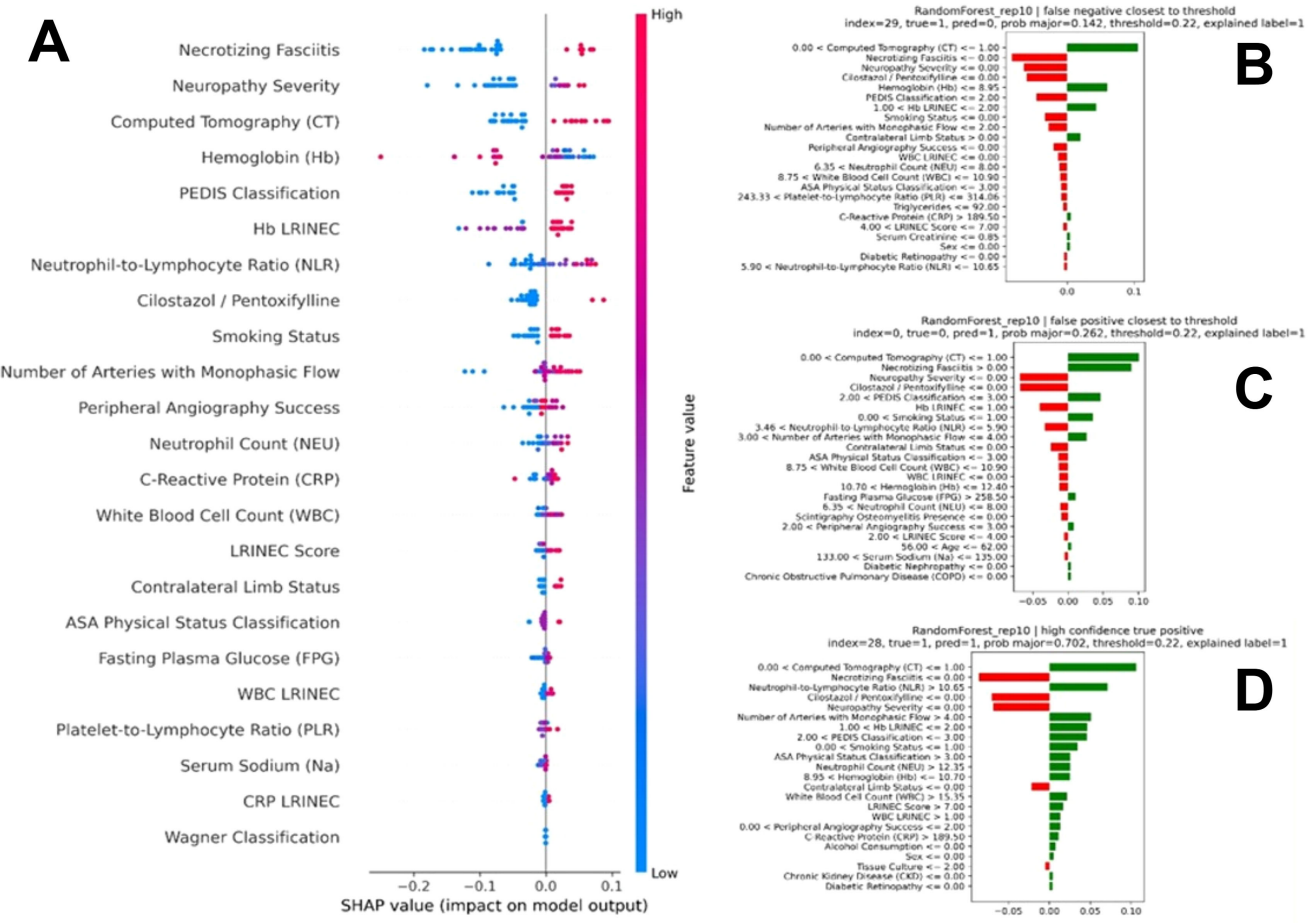
Explainable artificial intelligence outputs for the Random Forest model from repetition 11 in the external cohort. **(A)** SHAP beeswarm plot showing the global contribution of the selected features to the model output. **(B)** LIME explanation for the false negative case closest to the decision threshold. **(C)** LIME explanation for the false positive case closest to the decision threshold. **(D)** LIME explanation for a high-confidence true positive case.

**Figure 7** presents the global and local explanations of the Random Forest model on the external cohort. The SHAP beeswarm plot in **Figure 7A** shows that the variables contributing most strongly to the model output include Necrotizing Fasciitis, Neuropathy Severity, CT, Hb, PEDIS Classification, Hb-LRINEC, and NLR. In addition, variables such as Cilostazol/Pentoxifylline, Smoking Status, Number of Arteries with Monophasic Flow, and Peripheral Angiography Success also made meaningful contributions to the model output. **Figure 7B** shows the LIME explanation for the false negative case closest to the decision threshold. In this case, variables such as CT, Hb, and Hb-LRINEC contributed toward the major amputation class, whereas opposite contributions from variables including Necrotizing Fasciitis, Neuropathy Severity, Cilostazol/Pentoxifylline, and Contralateral Limb Status were associated with the final negative classification. **Figure 7C** presents the false positive case closest to the decision threshold. In this example, CT, Necrotizing Fasciitis, PEDIS Classification, Hb-LRINEC, and Smoking Status contributed toward the major amputation class, despite opposing contributions from several other variables. **Figure 7D** shows the LIME explanation for a high-confidence true positive case. In this example, CT, NLR, Number of Arteries with Monophasic Flow, Hb-LRINEC, PEDIS Classification, Smoking Status, ASA Physical Status Classification, and NEU were among the main contributors supporting the major amputation prediction.

## 4 Discussion

In this study, we developed and temporally tested a machine-learning framework to support preoperative prediction of whether an initially limb-preserving strategy in diabetic foot management would ultimately culminate in minor or major amputation. Several findings stand out. First, the major-amputation group exhibited a more severe baseline phenotype across inflammatory markers, LRINEC-related measures, functional status, neuropathic burden, and wound-severity classifications. Second, the embedded feature-selection procedure yielded a stable core predictor set across 20 repeated outer splits, suggesting that the learned signal was reproducible rather than split-specific. Third, no single classifier dominated every evaluation domain: MLP achieved the highest mean internal AP (55.9%), whereas KNN achieved the highest mean external AP (65.1%) and recall (72.8%); by contrast, MLP retained the highest external precision (56.2%) and specificity (82.9%), while Random Forest and KNN showed the most favorable external Brier profiles (both 0.157). Finally, the explainable AI analyses highlighted a clinically coherent group of drivers related to infection severity, neuropathic burden, systemic inflammation, and vascular compromise.

The exploratory analyses support the clinical validity of the prediction problem. In Dataset 1, the major-amputation group was characterized by higher WBC, NEU, CRP, NLR, PLR, and LRINEC-related measures, together with lower Hb and albumin. Severity-oriented clinical variables, including ASA, Wagner, PEDIS, and Neuropathy Severity, were also shifted toward worse categories in the same group. On the categorical side, lower ambulation status, more frequent CT use, more frequent Cilostazol/Pentoxifylline use, and a higher prevalence of Necrotizing Fasciitis were observed among patients who ultimately underwent major amputation. Taken together, these findings suggest that failure of an initially limb-preserving pathway is not driven by a single lesion characteristic, but rather by the co-occurrence of systemic inflammatory burden, advanced local infection and tissue destruction, neuropathic and functional impairment, and vascular disease. In Dataset 2, fewer between-group differences remained significant after multiple-testing correction, but the overall direction of effect broadly mirrored Dataset 1, which supports the view that the external cohort represented the same underlying clinical problem despite its smaller size.

That interpretation is consistent with the thresholding strategy used in the pipeline. Decision thresholds were tuned on a hold-out validation set by maximizing the F2-score under a minimum recall constraint of 0.70, which intentionally gives greater weight to avoiding false negatives for major amputation risk. Clinically, this reflects a preference not to underestimate the likelihood that an initially limb-preserving approach will fail. From this perspective, the stronger external recall observed for KNN is attractive in a safety-oriented setting, whereas the higher precision and specificity of MLP may be more desirable when unnecessary escalation to more proximal surgery is a major concern. The coexistence of these different strengths is therefore not a weakness of the study; rather, it highlights that predictive tools in diabetic foot surgery should be interpreted in relation to the downstream consequences of false-negative and false-positive errors.

Calibration adds another clinically important dimension to the discussion. A model may separate higher-risk from lower-risk patients reasonably well while still producing poorly calibrated probabilities. In the present study, calibration deteriorated overall in the external cohort, which is not unexpected when models are transferred to a smaller and distinct test population. Nevertheless, Random Forest and KNN remained closer to the diagonal over substantial portions of the calibration curve and showed the most favorable external Brier profiles, whereas MLP and XGBoost were less favorable in this respect. For a decision-support system intended to assist multidisciplinary planning, this distinction matters. When model output is used not only for ranking but also for communicating risk, prioritizing reassessment, or guiding preoperative vigilance, better-calibrated probabilities may be as important as discrimination performance.

The explainable AI findings further support the clinical coherence of the learned signal. Random Forest was selected as the primary interpretability model because it combined strong internal performance with a comparatively balanced external profile in both discrimination and calibration. In the external cohort, SHAP identified Necrotizing Fasciitis, Neuropathy Severity, CT, Hb, PEDIS Classification, Hb-LRINEC, and NLR among the strongest contributors to model output, with additional contributions from Cilostazol/Pentoxifylline use, smoking status, number of arteries with monophasic flow, and peripheral angiography success. This constellation of variables is clinically meaningful because it brings together markers of aggressive soft-tissue infection, advanced wound burden, neuropathic damage, systemic inflammatory stress, anemia-related vulnerability, and vascular insufficiency. The LIME examples are equally informative, showing that near-threshold false-negative and false-positive cases were driven by competing signals rather than by a single dominant feature. Accordingly, the XAI outputs should be interpreted as descriptions of how the model integrates correlated clinical cues, not as proof of causal effects of individual variables.

Clinically, this problem matters because both types of error carry substantial consequences. Overestimation of major amputation risk may contribute to unnecessarily proximal surgery, with loss of mobility and functional independence, whereas underestimation may prolong unsuccessful salvage attempts, delay definitive infection control, and increase the burden of repeated procedures, reamputation, hospitalization, and cost (9, 11, 12, 18, 19). For that reason, the most realistic role of a model such as this is not to replace clinical judgment, but to support multidisciplinary decision-making at the time of initial presentation, when limb salvage potential and the most distal feasible level of surgery are being weighed simultaneously (5, 6, 8, 10).

An additional practical point is that the present findings should not be framed as machine learning versus conventional bedside scoring. Conventional ulcer classification systems such as Wagner, PEDIS, and SINBAD remain useful for structured assessment, communication, and risk stratification, but they were developed for partly different purposes and vary in validation status across clinical use cases (26). Recent work has also shown that structured wound-classification systems continue to carry prognostic information for amputation risk (27, 28). This is consistent with the repeated retention of Wagner, PEDIS, and neuropathy-related features in our own pipeline. Accordingly, our data support complementarity rather than replacement: the incremental value of the machine-learning framework lies in integrating these structured clinical assessments with laboratory indices and selected imaging/vascular descriptors within a single predictive model. Because we did not perform a direct head-to-head benchmark against standalone Wagner, PEDIS, or SINBAD scores, the present study should not be interpreted as proving superiority over those systems.

These findings should be interpreted in the context of a growing body of AI research on diabetic foot amputation (20–23). Wang et al. developed machine-learning models to predict minor amputation in patients with University of Texas grade 3 diabetic foot ulcers (20). Stefanopoulos et al. reported a machine-learning model for amputation prediction in patients with diabetes (21), Demirkol et al. modeled amputation risk in a tertiary diabetic foot cohort (22), and Oei et al. developed explainable machine-learning models for diabetic foot amputation risk (23). Against that background, the main contribution of the present study is not the introduction of a novel algorithmic class, but the framing of a different and clinically practical question: among patients selected for an initial limb-preserving strategy, which cases are more likely to culminate in minor versus major amputation? In parallel, adjacent imaging-AI studies by Cuce et al. and Cakir et al. addressed diagnostic differentiation between osteomyelitis and Charcot neuroarthropathy rather than surgical outcome prediction and should therefore be regarded as related, but not directly comparable, literature (24, 25).

Several limitations should be acknowledged. First, the study was retrospective, and the observed associations may reflect local practice patterns in addition to disease biology. Second, class imbalance required SMOTE within the training pipeline; although oversampling was restricted to the training data, performance may shift under different class distributions. Third, the external cohort was considerably smaller than the development dataset, which likely contributed to wider variability, fewer statistically significant between-group differences in the exploratory analyses, and overall deterioration in calibration. Fourth, the analysis was conducted at the patient level, with one preoperative sample per patient; generalizability should nevertheless be interpreted in light of the retrospective single-center design and the temporal split within the same overarching clinical population. An additional limitation is the absence of consistent information regarding the timing of major amputation. The model treats patients progressing to major amputation shortly after the initial limb-preserving procedure and those progressing months or years later as a single endpoint. Clinically, however, these scenarios likely represent distinct disease trajectories. Early failure is more likely to reflect baseline tissue destruction, severe infection, necrotizing fasciitis, and acute inflammatory burden, whereas late failure may additionally be influenced by recurrent ulceration, recurrent infection, vascular restenosis, adherence to off-loading, and the quality of long-term multidisciplinary preventive care. Because these temporal data were not uniformly available in this retrospective cohort, time-to-event analyses could not be performed reliably. Consequently, the proposed model should be interpreted primarily as a baseline preoperative risk-stratification tool to support the initial surgical decision-making process, rather than as a longitudinal survival prediction model. Future prospective studies should systematically capture the interval between the initial limb-preserving procedure and major amputation, enabling the development and validation of time-to-event, multi-state, or survival-based machine-learning models.

Another important limitation is the insufficient characterization of baseline ischemic severity using standardized vascular indices. Established parameters such as the Ankle-Brachial Index (ABI), Toe-Brachial Index (TBI), and the Society for Vascular Surgery WIfI classification were not consistently recorded across this retrospective cohort, while specialized perfusion assessment methods such as transcutaneous oxygen pressure (TcPO_2_) and skin perfusion pressure (SPP) were not routinely available at our institution during the study period. To avoid substantial missing data and potential selection bias, these variables were not incorporated into the machine-learning analysis. Instead, the vascular component of the model relied on consistently available variables, including the number of lower extremity arteries with monophasic flow and peripheral angiography success. Consequently, although the model incorporated routinely available vascular information, the absence of standardized hemodynamic and tissue perfusion measurements limits the interpretation of the independent contribution of ischemia to the prediction of major amputation. Future prospective studies should incorporate standardized vascular assessment, including ABI, TBI, TcPO_2_, SPP, and WIfI classification, to better characterize ischemic burden and further improve model interpretability and generalizability.

## 5 Conclusions

In conclusion, the present findings suggest that prediction of minor versus major amputation after an initially limb-preserving strategy in diabetic foot is feasible using routinely available baseline clinical, laboratory, and selected imaging/vascular data. The underlying signal was stable, temporally validated, and clinically interpretable, but no single classifier dominated across all performance domains. Future work should focus on prospective validation, broader external testing, explicit benchmarking against established clinical scores, and recalibration before implementation in routine practice. In that setting, the most realistic role of such a model is not to replace the surgeon or radiologist, but to support multidisciplinary decision-making in complex diabetic foot management.

## Data Availability

The original contributions presented in the study are included in the article/**Supplementary Material**. Further inquiries can be directed to the corresponding authors.
