# Supplementary material for: Machine learning–based prediction of major amputation risk after initial limb-preserving surgery in diabetic foot
Source: Front Endocrinol (Lausanne). 2026 Aug 3;17:1889329. doi: 10.3389/fendo.2026.1889329 (PMC13478262; doi:10.3389/fendo.2026.1889329)
Supplement: Supplementary file 1 [file DataSheet1.zip › supplementary material 1v2 1-1.pdf]

Supplementary Table S1. Dataset 1 Group-wise distribution and statistical comparison of continuous (cont.) and ordinal (ord.) demographic, laboratory, and clinical variables between Class 0 and Class 1 (mean±SD; median [IQR]), including primary p-values and multiple-comparison–adjusted p-values (Benjamini–Hochberg FDR and Holm–Bonferroni) with effect sizes (Cohen's d, Cliff's delta).

Feature	Type	n class0	n class1	Mean±SD class0	Mean±SD class1	Median (IQR) class0	Median (IQR) class1	Delta mean (1-0)	Welch t p two sided	Mann–Whitney U p two sided	Levene p	Cohen d	Cliffs delta	p primary	test_primary	p FDR BH	p Holm	sig FDR < 0.01
Age	cont.	538	55	63.73 ± 10.54	65.56 ± 10.88	64.00 (57.00–71.00)	66.00 (59.50–74.00)	1.83	0.24	0.09	0.75	0.17	0.14	0.24	Welch t	0.32	1.00	0
Educational Status	ord.	538	55	2.70 ± 1.18	2.29 ± 1.21	3.00 (2.00–4.00)	2.00 (2.00–3.00)	-0.41		0.01		-0.35	-0.21	0.01	Mann–Whitney U	0.01	0.15	0
Height	cont.	538	55	170.20 ± 7.38	168.04 ± 7.67	170.00 (165.00–175.00)	170.00 (160.00–175.00)	-2.17	0.05	0.08	0.37	-0.29	-0.14	0.05	Welch t	0.08	0.79	0
Weight	cont.	538	55	78.28 ± 11.43	74.91 ± 9.82	80.00 (70.00–82.00)	79.00 (70.00–82.00)	-3.37	0.02	0.05	0.63	-0.30	-0.16	0.02	Welch t	0.04	0.40	0
Body Mass	con	538	55	27.08	26.53	26.80 (24.	26.80 (24.	-0.55	0.29	0.33	0.	-0.	-0.	0.29	Welch t	0.35	1.	0

Index (BMI)	t.			± 3.90	± 3.60	20–28.40)	20–27.65)				90	14	08				00	
Fasting Plasma Glucose (FPG)	cont.	538	55	189.22 ± 96.87	233.75 ± 134.07	169.50 (123.25–229.75)	198.00 (145.00–283.50)	44.53	0.02	0.02	0.01	0.44	0.19	0.02	Mann–Whitney U	0.04	0.40	0
FPG LRINEC	ord.	538	55	0.46 ± 0.50	0.55 ± 0.50	0.00 (0.00–1.00)	1.00 (0.00–1.00)	0.08		0.25		0.16	0.08	0.25	Mann–Whitney U	0.33	1.00	0
White Blood Cell Count (WBC)	cont.	538	55	10.67 ± 4.80	15.15 ± 6.65	9.60 (7.50–12.38)	14.00 (10.10–18.20)	4.48	<0.01	<0.01	<0.01	0.90	0.44	<0.01	Mann–Whitney U	<0.01	<0.01	1
WBC LRINEC	ord.	538	55	0.17 ± 0.43	0.56 ± 0.71	0.00 (0.00–0.00)	0.00 (0.00–1.00)	0.39		<0.01		0.86	0.30	<0.01	Mann–Whitney U	<0.01	<0.01	1
Hemoglobin (Hb)	cont.	538	55	11.53 ± 4.63	9.72 ± 1.46	11.30 (10.10–12.60)	9.80 (8.70–10.90)	-1.81	<0.01	<0.01	0.35	-0.41	-0.49	<0.01	Welch t	<0.01	<0.01	1
Hb LRINEC	ord.	538	55	1.30 ± 0.72	1.76 ± 0.43	1.00 (1.00–	2.00 (2.00–	0.46		<0.01		0.66	0.34	<0.01	Mann–Whit	<0.01	<0.01	1

						2.00)	2.00)								ney U			
Neutrophil Count (NEU)	cont.	538	55	7.73 ± 4.39	12.56 ± 6.33	6.70 (4.90–9.20)	11.40 (7.85–14.95)	4.82	<0.01	<0.01	<0.01	1.05	0.51	<0.01	Mann–Whitney U	<0.01	<0.01	1
Platelet Count (PLT)	cont.	538	55	339.77 ± 128.80	360.55 ± 129.31	320.00 (244.25–411.75)	358.00 (280.50–445.50)	20.78	0.26	0.11	0.76	0.16	0.13	0.26	Welch t	0.33	1.00	0
Lymphocyte Count (LYM)	cont.	538	55	1.86 ± 0.72	1.55 ± 1.60	1.70 (1.40–2.27)	1.30 (0.90–1.65)	-0.31	0.16	<0.01	0.16	-0.37	-0.42	0.16	Welch t	0.23	1.00	0
C-Reactive Protein (CRP)	cont.	538	55	72.68 ± 82.02	139.94 ± 88.13	39.00 (10.00–108.00)	141.00 (57.50–211.50)	67.26	<0.01	<0.01	0.09	0.81	0.47	<0.01	Welch t	<0.01	<0.01	1
CRP LRINEC	ord.	538	55	0.67 ± 1.49	1.96 ± 2.02	0.00 (0.00–0.00)	0.00 (0.00–4.00)	1.29		<0.01		0.84	0.32	<0.01	Mann–Whitney U	<0.01	<0.01	1
Serum Sodium (Na)	cont.	538	55	136.34 ± 3.71	133.67 ± 5.08	137.00 (134.00–139.00)	135.00 (131.50–138.00)	-2.67	<0.01	<0.01	<0.01	-0.69	-0.30	0.00	Mann–Whitney U	0.00	0.01	1

Na LRINEC	ord.	538	55	0.52 ± 0.88	0.91 ± 1.01	0.00 (0.00–2.00)	0.00 (0.00–2.00)	0.38		<0.01		0.43	0.19	<0.01	Mann–Whitney U	0.01	0.06	1
Blood Urea Nitrogen (BUN)	cont.	538	55	52.84 ± 34.94	60.49 ± 30.46	45.00 (34.00–64.00)	51.00 (39.00–78.00)	7.65	0.08	0.04	0.44	0.22	0.17	0.08	Welch t	0.13	1.00	0
Serum Creatinine	cont.	538	55	1.31 ± 1.08	1.42 ± 1.27	1.00 (0.80–1.35)	1.00 (0.88–1.40)	0.11	0.55	0.62	0.51	0.10	0.04	0.55	Welch t	0.60	1.00	0
Creatinine LRINEC	ord.	538	55	0.28 ± 0.70	0.36 ± 0.78	0.00 (0.00–0.00)	0.00 (0.00–0.00)	0.08		0.42		0.12	0.04	0.42	Mann–Whitney U	0.48	1.00	0
Glomerular Filtration Rate (GFR)	cont.	538	55	73.13 ± 53.00	68.53 ± 34.73	70.50 (51.00–90.00)	64.00 (48.00–88.50)	-4.61	0.38	0.33	0.98	-0.09	-0.08	0.38	Welch t	0.45	1.00	0
Neutrophil-to-Lymphocyte Ratio (NLR)	cont.	538	55	4.99 ± 4.30	12.73 ± 11.99	3.73 (2.47–6.12)	9.57 (5.76–14.95)	7.74	<0.01	<0.01	<0.01	1.41	0.57	<0.01	Mann–Whitney U	<0.01	<0.01	1

Platelet-to-Lymphocyte Ratio (PLR)	cont.	538	55	208.23 ± 112.96	322.81 ± 211.45	185.52 (130.36–260.56)	277.50 (197.14–386.42)	114.59	<0.01	<0.01	<0.01	0.92	0.40	<0.01	Mann–Whitney U	<0.01	<0.01	1
Erythrocyte Sedimentation Rate (ESR)	cont.	538	55	71.41 ± 32.79	81.04 ± 32.98	75.00 (46.00–93.00)	78.00 (61.50–106.00)	9.62	0.04	0.08	0.86	0.29	0.14	0.04	Welch t	0.07	0.73	0
Serum Albumin	cont.	538	55	3.56 ± 1.81	3.06 ± 0.51	3.50 (3.10–3.90)	3.10 (2.65–3.45)	-0.50	<0.01	<0.01	0.71	-0.29	-0.44	<0.01	Welch t	<0.01	<0.01	1
Triglycerides	cont.	538	55	154.76 ± 93.60	169.05 ± 139.79	134.00 (100.25–180.00)	141.00 (100.00–188.50)	14.29	0.46	0.65	0.33	0.14	0.04	0.46	Welch t	0.51	1.00	0
Tissue Culture	ord.	538	55	2.13 ± 0.68	2.31 ± 0.60	2.00 (2.00–3.00)	2.00 (2.00–3.00)	0.18		0.07		0.27	0.13	0.07	Mann–Whitney U	0.11	1.00	0
Hemoglobin A1c (HbA1c)	cont.	538	55	9.40 ± 2.29	9.25 ± 2.77	9.10 (7.70–10.70)	8.70 (7.10–11.20)	-0.14	0.71	0.31	0.07	-0.06	-0.08	0.71	Welch t	0.73	1.00	0
Contralateral Limb Status	ord.	538	55	0.20 ±	0.47 ±	0.00 (0.00–	0.00 (0.00–	0.27		0.01		0.40	0.11	0.01	Mann–W	0.02	0.23	0

				0.65	1.03	0.00)	0.00)								hitney U			
Number of Arteries with Monophasic Flow	cont.	538	55	1.86 ± 1.51	2.95 ± 1.37	2.00 (0.00–3.00)	3.00 (2.00–4.00)	1.09	<0.01	<0.01	0.16	0.73	0.40	<0.01	Welch t	<0.01	<0.01	1
MRI Osteomyelitis Presence	ord.	538	55	0.46 ± 1.01	0.45 ± 0.81	0.00 (0.00–0.00)	0.00 (0.00–0.50)	-0.01		0.59		-0.01	0.03	0.59	Mann–Whitney U	0.62	1.00	0
Scintigraphy Osteomyelitis Presence	ord.	538	55	0.95 ± 1.33	0.75 ± 1.04	0.00 (0.00–3.00)	0.00 (0.00–1.00)	-0.20		1.00		-0.16	0.00	1.00	Mann–Whitney U	1.00	1.00	0
Peripheral Angiography Success	ord.	538	55	1.86 ± 1.29	2.29 ± 0.81	2.00 (0.00–3.00)	2.00 (2.00–3.00)	0.44		0.17		0.35	0.10	0.17	Mann–Whitney U	0.24	1.00	0
ASA Physical Status Classification	ord.	538	55	2.86 ± 0.47	3.11 ± 0.42	3.00 (3.00–3.00)	3.00 (3.00–3.00)	0.25		<0.01		0.53	0.22	<0.01	Mann–Whitney U	<0.01	0.01	1
Wagner	or	538	55	3.83 ±	4.02 ±	4.00 (4.0	4.00 (4.0	0.19		<0.01		0.	0.18	<0.	Mann–	<0.	0.	1

Classification	d.			0.38	0.30	0–4.00)	0–4.00)					50		01	Whitney U	01	02	
PEDIS Classification	ord.	538	55	2.46 ± 0.50	2.89 ± 0.37	2.00 (2.00–3.00)	3.00 (3.00–3.00)	0.43		<0.01		0.88	0.42	<0.01	Mann–Whitney U	<0.01	<0.01	1
LRINEC Score	ord.	538	55	3.41 ± 2.68	6.11 ± 3.41	3.00 (1.00–5.00)	6.00 (3.50–9.50)	2.70		<0.01		0.98	0.47	<0.01	Mann–Whitney U	<0.01	<0.01	1
Neuropathy Severity	ord.	538	55	1.02 ± 1.16	1.89 ± 1.05	1.00 (0.00–2.00)	2.00 (1.00–3.00)	0.87		<0.01		0.75	0.42	<0.01	Mann–Whitney U	<0.01	<0.01	1

Supplementary Table S2. Dataset 1 Group-wise distribution and statistical comparison of categorical variables (binary and nominal) between Class 0 and Class 1, reported as n (%). Pearson's chi-square test was used by default, and Fisher's exact test was applied for 2×2 tables with small expected cell counts; primary p-values were adjusted using Benjamini–Hochberg FDR and Holm–Bonferroni procedures.

**Feature**	**Type**	**Level**	**class 0 n (%)**	**class 1 n (%)**	**Chi-square p**	**Fisher p**	**p primary**	**test_primary**	**p FDR BH**	**p Holm**	**sig FDR <0.01**
Sex	binary	0	420 (78.1%)	38 (69.1%)	0.18		0.18	Chi-square	0.54	1.00	0

Sex	binary	1	118 (21.9%)	17 (30.9%)							
Marital Status	binary	0	149 (27.7%)	12 (21.8%)	0.44		0.44	Chi-square	0.91	1.00	0
Marital Status	binary	1	389 (72.3%)	43 (78.2%)							
Place of Residence	binary	0	100 (18.6%)	8 (14.5%)	0.58		0.58	Chi-square	0.93	1.00	0
Place of Residence	binary	1	438 (81.4%)	47 (85.5%)							
Smoking Status	binary	0	385 (71.6%)	30 (54.5%)	0.01		0.01	Chi-square	0.08	0.34	0
Smoking Status	binary	1	153 (28.4%)	25 (45.5%)							
Alcohol Consumption	binary	0	493 (91.6%)	51 (92.7%)	0.98	1.00	1.00	Fisher	1.00	1.00	0
Alcohol Consumption	binary	1	45 (8.4%)	4 (7.3%)							
Family History	binary	0	395 (73.4%)	38 (69.1%)	0.60		0.60	Chi-square	0.93	1.00	0
Family History	binary	1	143 (26.6%)	17 (30.9%)							
Ambulation Status	binary	0	71 (13.2%)	21 (38.2%)	<0.01		<0.01	Chi-square	<0.01	<0.01	1
Ambulation Status	binary	1	467 (86.8%)	34 (61.8%)							
Type of Footwear	binary	0	143 (26.6%)	16 (29.1%)	0.81		0.81	Chi-square	1.00	1.00	0
Type of Footwear	binary	1	395 (73.4%)	39 (70.9%)							

Insulin Use	binary	0	159 (29.6%)	11 (20.0%)	0.18		0.18	Chi-square	0.54	1.00	0
Insulin Use	binary	1	379 (70.4%)	44 (80.0%)							
Oral Antidiabetic Drug (OAD) Use	binary	0	222 (41.3%)	28 (50.9%)	0.22		0.22	Chi-square	0.57	1.00	0
Oral Antidiabetic Drug (OAD) Use	binary	1	316 (58.7%)	27 (49.1%)							
Hypertension (HT)	binary	0	207 (38.5%)	21 (38.2%)	1.00		1.00	Chi-square	1.00	1.00	0
Hypertension (HT)	binary	1	331 (61.5%)	34 (61.8%)							
Coronary Artery Disease (CAD)	binary	0	279 (51.9%)	26 (47.3%)	0.61		0.61	Chi-square	0.93	1.00	0
Coronary Artery Disease (CAD)	binary	1	259 (48.1%)	29 (52.7%)							
Chronic Kidney Disease (CKD)	binary	0	369 (68.6%)	43 (78.2%)	0.19		0.19	Chi-square	0.54	1.00	0
Chronic Kidney Disease (CKD)	binary	1	169 (31.4%)	12 (21.8%)							
Chronic Obstructive Pulmonary Disease (COPD)	binary	0	505 (93.9%)	52 (94.5%)	1.00	1.00	1.00	Fisher	1.00	1.00	0
Chronic Obstructive Pulmonary Disease (COPD)	binary	1	33 (6.1%)	3 (5.5%)							
Peripheral Arterial Disease (PAD)	binary	0	428 (79.6%)	51 (92.7%)	0.03		0.03	Chi-square	0.14	0.70	0
Peripheral Arterial Disease (PAD)	binary	1	110 (20.4%)	4 (7.3%)							
Acetylsalicylic Acid (ASA use)	binary	0	270 (50.2%)	19 (34.5%)	0.04		0.04	Chi-square	0.16	0.89	0

Acetylsalicylic Acid (ASA use)	binary	1	268 (49.8 %)	36 (65.5 %)							
Cilostazol / Pentoxifylline	binary	0	496 (92.2 %)	36 (65.5 %)	<0.01		<0.01	Chi-square	<0.01	<0.01	1
Cilostazol / Pentoxifylline	binary	1	42 (7.8 %)	19 (34.5 %)							
NOAH	binary	0	500 (92.9 %)	51 (92.7 %)	1.00	1.00	1.00	Fisher	1.00	1.00	0
NOAH	binary	1	38 (7.1 %)	4 (7.3 %)							
Computed Tomography (CT)	binary	0	417 (77.5 %)	29 (52.7 %)	<0.01		<0.01	Chi-square	<0.01	<0.01	1
Computed Tomography (CT)	binary	1	121 (22.5 %)	26 (47.3 %)							
Magnetic Resonance Imaging (MRI)	binary	0	428 (79.6 %)	41 (74.5 %)	0.49		0.49	Chi-square	0.93	1.00	0
Magnetic Resonance Imaging (MRI)	binary	1	110 (20.4 %)	14 (25.5 %)							
Peripheral Angiography	binary	0	156 (29.0 %)	18 (32.7 %)	0.67		0.67	Chi-square	0.93	1.00	0
Peripheral Angiography	binary	1	382 (71.0 %)	37 (67.3 %)							
Necrotizing Fasciitis	binary	0	387 (71.9 %)	15 (27.3 %)	<0.01		<0.01	Chi-square	<0.01	<0.01	1
Necrotizing Fasciitis	binary	1	151 (28.1 %)	40 (72.7 %)							
Diabetic Nephropathy	binary	0	369 (68.6 %)	40 (72.7 %)	0.63		0.63	Chi-square	0.93	1.00	0
Diabetic Nephropathy	binary	1	169 (31.4 %)	15 (27.3 %)							

Diabetic Retinopathy	binary	0	410 (76.2 %)	44 (80.0 %)	0.64		0.64	Chi-square	0.93	1.00	0
Diabetic Retinopathy	binary	1	128 (23.8 %)	11 (20.0 %)							
Dialysis Status	binary	0	503 (93.5 %)	51 (92.7 %)	1.00	0.78	0.78	Fisher	1.00	1.00	0
Dialysis Status	binary	1	35 (6.5 %)	4 (7.3 %)							
Negative Pressure Wound Therapy	binary	0	313 (58.2 %)	27 (49.1 %)	0.25		0.25	Chi-square	0.60	1.00	0
Negative Pressure Wound Therapy	binary	1	225 (41.8 %)	28 (50.9 %)							
Physiotherapy	binary	0	125 (23.2 %)	17 (30.9 %)	0.27		0.27	Chi-square	0.60	1.00	0
Physiotherapy	binary	1	413 (76.8 %)	38 (69.1 %)							

Supplementary Table S3. Dataset 2 Group-wise distribution and statistical comparison of cont. and ord. demographic, laboratory, and clinical variables between Class 0 and Class 1 (mean±SD; median [IQR]), including primary p-values, test selection (Welch's t-test vs Mann–Whitney U), multiple-comparison corrections (Benjamini–Hochberg FDR and Holm–Bonferroni), and effect sizes (Cohen's d and Cliff's delta).

**Feature**	**Type**	**n class0**	**n class1**	**Mean±SD class0**	**Mean±SD class1**	**Median (IQR) class0**	**Median (IQR) class1**	**Delta mean (1-0)**	**Welch t p two sided**	**Mann–Whitney U p two sided**	**Levene p**	**Cohen d**	**Cliffs delta**	**p primary**	**test_primary**	**p FDR BH**	**p Holm**	**sig FDR < 0.01**
Age	cont.	26	9	62.69 ± 15	61.89 ± 12	63.00 (57.50–73.50)	58.00 (53.00–75.00)	-0.80	0.88	0.35	0.80	-0.05	-0.21	0.88	Welch t	0.94	1.00	0

				.76	.05													
Educational Status	ord.	26	9	2.88 ± 1.03	2.78 ± 0.83	3.00 (2.00–3.00)	3.00 (2.00–3.00)	-0.11		0.78		-0.11	-0.06	0.78	Mann–Whitney U	0.92	1.00	0
Height	cont.	26	9	168.92 ± 8.30	168.22 ± 5.74	170.00 (161.25–175.00)	168.00 (165.00–170.00)	-0.70	0.78	0.57	0.31	-0.09	-0.13	0.78	Welch t	0.92	1	0
Weight	cont.	26	9	75.58 ± 12.97	77.00 ± 19.13	74.00 (70.00–82.00)	70.00 (65.00–75.00)	1.42	0.84	0.78	0.68	0.10	-0.07	0.84	Welch t	0.94	1.00	0
Body Mass Index (BMI)	cont.	26	9	26.52 ± 5.05	27.08 ± 5.98	24.95 (23.90–27.70)	24.50 (23.90–27.50)	0.56	0.80	1.00	0.83	0.11	0.00	0.80	Welch t	0.92	1.00	0
Fasting Plasma Glucose (FPG)	cont.	26	9	191.46 ± 72.30	269.78 ± 99.21	167.50 (156.25–231.50)	263.00 (229.00–300.00)	78.32	0.05	0.05	0.38	0.98	0.45	0.05	Welch t	0.16	1.00	0
FPG LRINEC	ord.	26	9	0.38 ± 0.50	0.78 ± 0.44	0.00 (0.00–1.00)	1.00 (1.00–1.00)	0.39		0.05		0.81	0.39	0.05	Mann–Whitney U	0.15	1.00	0

White Blood Cell Count (WBC)	cont.	26	9	11.37 ± 4.22	17.78 ± 10.25	10.75 (7.88–14.52)	15.50 (9.40–18.80)	6.41	0.10	0.07	0.05	1.03	0.41	0.07	Mann–Whitney U	0.18	1.00	0
WBC LRINEC	ord.	26	9	0.19 ± 0.40	0.78 ± 0.83	0.00 (0.00–0.00)	1.00 (0.00–1.00)	0.59		0.02		1.09	0.41	0.02	Mann–Whitney U	0.12	0.82	0
Hemoglobin (Hb)	cont.	26	9	11.11 ± 1.99	9.18 ± 1.51	11.25 (9.35–12.50)	8.80 (8.80–9.30)	-1.93	0.01	0.01	0.13	-1.02	-0.59	0.01	Welch t	0.06	0.25	0
Hb LRINEC	ord.	26	9	1.35 ± 0.69	1.89 ± 0.33	1.00 (1.00–2.00)	2.00 (2.00–2.00)	0.54		0.03		0.87	0.44	0.03	Mann–Whitney U	0.12	0.89	0
Neutrophil Count (NEU)	cont.	26	9	8.52 ± 3.97	15.44 ± 10.04	7.65 (5.47–11.22)	13.30 (7.50–16.80)	6.93	0.08	0.03	0.03	1.15	0.50	0.03	Mann–Whitney U	0.12	0.87	0
Platelet Count (PLT)	cont.	26	9	334.23 ± 135.27	394.89 ± 100.92	305.00 (279.50–353.75)	366.00 (334.00–486.00)	60.66	0.17	0.12	0.93	0.47	0.36	0.17	Welch t	0.35	1.00	0

Lymphocyte Count (LYM)	cont.	26	9	1.78 ± 0.90	1.26 ± 0.40	1.55 (1.23–2.20)	1.20 (0.90–1.50)	-0.53	0.02	0.11	0.15	-0.66	-0.37	0.02	Welch t	0.12	0.78	0
C-Reactive Protein (CRP)	cont.	26	9	99.33 ± 78.96	196.33 ± 68.57	104.00 (16.00–150.50)	208.00 (144.00–252.00)	97.00	<0.01	<0.01	0.49	1.27	0.65	<0.01	Welch t	0.04	0.11	0
CRP LRINEC	ord.	26	9	1.08 ± 1.81	2.67 ± 2.00	0.00 (0.00–3.00)	4.00 (0.00–4.00)	1.59		0.04		0.86	0.40	0.04	Mann–Whitney U	0.15	1.00	0
Serum Sodium (Na)	cont.	26	9	136.88 ± 4.00	133.33 ± 2.74	137.00 (134.00–140.00)	132.00 (131.00–135.00)	-3.55	0.01	0.03	0.17	-0.95	-0.50	0.01	Welch t	0.06	0.27	0
Na LRINEC	ord.	26	9	0.62 ± 0.94	1.11 ± 1.05	0.00 (0.00–2.00)	2.00 (0.00–2.00)	0.50		0.20		0.51	0.25	0.20	Mann–Whitney U	0.35	1.00	0
Blood Urea Nitrogen (BUN)	cont.	26	9	54.31 ± 20.87	95.67 ± 78.91	47.50 (40.00–67.25)	75.00 (39.00–96.00)	41.36	0.16	0.21	0.01	0.96	0.29	0.21	Mann–Whitney U	0.35	1.00	0

Serum Creatinine	cont.	26	9	1.10 ± 0.36	1.73 ± 1.20	1.10 (0.83–1.30)	1.30 (0.90–2.10)	0.64	0.15	0.25	0.01	0.95	0.26	0.25	Mann–Whitney U	0.36	1.00	0
Creatinine LRINEC	ord.	26	9	0.15 ± 0.54	0.67 ± 1.00	0.00 (0.00–0.00)	0.00 (0.00–2.00)	0.51		0.07		0.75	0.26	0.07	Mann–Whitney U	0.18	1.00	0
Glomerular Filtration Rate (GFR)	cont.	26	9	72.54 ± 26.35	58.56 ± 33.79	69.00 (52.25–89.75)	53.00 (34.00–89.00)	-13.98	0.28	0.28	0.33	-0.49	-0.25	0.28	Welch t	0.38	1.00	0
Neutrophil-to-Lymphocyte Ratio (NLR)	cont.	26	9	6.20 ± 5.22	14.39 ± 13.34	4.61 (2.74–7.16)	11.08 (6.25–15.00)	8.19	0.11	0.01	0.09	1.03	0.60	0.11	Welch t	0.23	1.00	0
Platelet-to-Lymphocyte Ratio (PLR)	cont.	26	9	230.62 ± 144.72	332.96 ± 114.96	205.95 (121.44–285.71)	305.00 (271.11–360.00)	102.34	0.05	0.03	0.31	0.74	0.50	0.05	Welch t	0.15	1.00	0
Erythrocyte Sedimentation Rate (ESR)	cont.	26	9	71.27 ± 41.10	95.44 ± 30.83	76.00 (38.25–97.50)	86.00 (72.00–116.00)	24.18	0.08	0.12	0.25	0.62	0.36	0.08	Welch t	0.18	1.00	0

Serum Albumin	cont.	26	9	3.55 ± 0.45	3.13 ± 0.27	3.65 (3.20–3.88)	3.20 (3.00–3.30)	-0.41	<0.01	0.01	0.11	-1.00	-0.56	<0.01	Welch t	0.04	0.12	0
Triglycerides	cont.	26	9	146.65 ± 86.65	120.44 ± 30.27	117.50 (89.50–151.00)	112.00 (108.00–134.00)	-26.21	0.19	0.94	0.19	-0.34	-0.02	0.19	Welch t	0.35	1.00	0
Tissue Culture	ord.	26	9	2.00 ± 0.69	2.33 ± 0.71	2.00 (2.00–2.00)	2.00 (2.00–3.00)	0.33		0.22		0.48	0.26	0.22	Mann–Whitney U	0.35	1.00	0
Hemoglobin A1c (HbA1c)	cont.	26	9	9.19 ± 2.19	9.03 ± 1.31	8.95 (7.15–10.85)	8.80 (8.20–10.20)	-0.16	0.80	0.89	0.07	-0.08	0.03	0.80	Welch t	0.92	1.00	0
Contralateral Limb Status	ord.	26	9	0.27 ± 0.78	0.56 ± 0.88	0.00 (0.00–0.00)	0.00 (0.00–1.00)	0.29		0.20		0.36	0.20	0.20	Mann–Whitney U	0.35	1.00	0
Number of Arteries with Monophasic Flow	cont.	26	9	2.92 ± 1.47	3.00 ± 1.41	3.00 (2.00–4.00)	3.00 (2.00–3.00)	0.08	0.89	0.71	0.63	0.05	-0.09	0.89	Welch t	0.94	1.00	0
MRI Osteomyelitis	ord.	26	9	0.38 ±	0.78 ±	0.00 (0.00–	0.00 (0.00–	0.39		0.22		0.48	0.22	0.22	Mann–W	0.35	1.00	0

Presence				0.75	0.97	0.00)	2.00)								hitney U			
Scintigraphy Osteomyelitis Presence	ord.	26	9	0.46 ± 1.10	0.00 ± 0.00	0.00 (0.00–0.00)	0.00 (0.00–0.00)	-0.46		0.23		-0.48	-0.15	0.23	Mann–Whitney U	0.35	1.00	0
Peripheral Angiography Success	ord.	26	9	1.42 ± 1.30	1.44 ± 1.42	1.50 (0.00–3.00)	2.00 (0.00–3.00)	0.02		1.00		0.02	0.00	1.00	Mann–Whitney U	1.00	1.00	0
ASA Physical Status Classification	ord.	26	9	2.96 ± 0.20	3.11 ± 0.60	3.00 (3.00–3.00)	3.00 (3.00–3.00)	0.15		0.27		0.44	0.14	0.27	Mann–Whitney U	0.38	1.00	0
Wagner Classification	ord.	26	9	4.00 ± 0.00	4.00 ± 0.00	4.00 (4.00–4.00)	4.00 (4.00–4.00)	0.00		1.00		0.00	0.00	1.00	Mann–Whitney U	1.00	1.00	0
PEDIS Classification	ord.	26	9	2.42 ± 0.50	2.78 ± 0.44	2.00 (2.00–3.00)	3.00 (3.00–3.00)	0.35		0.07		0.72	0.35	0.07	Mann–Whitney U	0.18	1.00	0

LRINEC Score	ord.	26	9	3.77 ± 2.55	7.89 ± 3.18	3.50 (2.00–5.75)	9.00 (5.00–9.00)	4.12		<0.01		1.52	0.70	<0.01	Mann–Whitney U	0.04	0.08	0
Neuropathy Severity	ord.	26	9	0.54 ± 0.99	0.78 ± 1.09	0.00 (0.00–0.75)	0.00 (0.00–1.00)	0.24		0.42		0.24	0.15	0.42	Mann–Whitney U	0.53	1.00	0

Supplementary Table S4. Dataset 2 Group-wise distribution and statistical comparison of categorical variables (binary and nominal) between Class 0 and Class 1, reported as n (%). Pearson’s chi-square test was used by default, and Fisher’s exact test was applied for 2×2 tables with small expected cell counts; primary p-values were adjusted using Benjamini–Hochberg FDR and Holm–Bonferroni procedures.

**Feature**	**Type**	**Level**	**class 0 n (%)**	**class 1 n (%)**	**Chi-square p**	**Fisher p**	**p primary**	**test_primary**	**p FDR BH**	**p Holm**	**sig FDR <0.01**
Sex	binary	0	17 (65.4%)	7 (77.8%)	0.78	0.69	0.69	Fisher	0.90	1.00	0
Sex	binary	1	9 (34.6%)	2 (22.2%)							
Marital Status	binary	0	6 (23.1%)	3 (33.3%)	0.87	0.66	0.66	Fisher	0.90	1.00	0
Marital Status	binary	1	20 (76.9%)	6 (66.7%)							
Place of Residence	binary	0	7 (26.9%)	1 (11.1%)	0.61	0.65	0.65	Fisher	0.90	1.00	0
Place of Residence	binary	1	19 (73.1%)	8 (88.9%)							

Smoking Status	binary	0	15 (57.7%)	6 (66.7%)	0.94	0.71	0.71	Fisher	0.90	1.00	0
Smoking Status	binary	1	11 (42.3%)	3 (33.3%)							
Alcohol Consumption	binary	0	24 (92.3%)	8 (88.9%)	1.00	1.00	1.00	Fisher	1.00	1.00	0
Alcohol Consumption	binary	1	2 (7.7%)	1 (11.1%)							
Family History	binary	0	22 (84.6%)	6 (66.7%)	0.50	0.34	0.34	Fisher	0.88	1.00	0
Family History	binary	1	4 (15.4%)	3 (33.3%)							
Ambulation Status	binary	1	26 (100.0%)	9 (100.0%)	1.00		1.00	Chi-square	1.00	1.00	0
Type of Footwear	binary	0	9 (34.6%)	4 (44.4%)	0.90	0.70	0.70	Fisher	0.90	1.00	0
Type of Footwear	binary	1	17 (65.4%)	5 (55.6%)							
Insulin Use	binary	0	6 (23.1%)	4 (44.4%)	0.43	0.39	0.39	Fisher	0.88	1.00	0
Insulin Use	binary	1	20 (76.9%)	5 (55.6%)							
Oral Antidiabetic Drug (OAD) Use	binary	0	10 (38.5%)	1 (11.1%)	0.27	0.22	0.22	Fisher	0.88	1.00	0
Oral Antidiabetic Drug (OAD) Use	binary	1	16 (61.5%)	8 (88.9%)							
Hypertension (HT)	binary	0	8 (30.8%)	1 (11.1%)	0.47	0.39	0.39	Fisher	0.88	1.00	0
Hypertension (HT)	binary	1	18 (69.2%)	8 (88.9%)							

Coronary Artery Disease (CAD)	binary	0	12 (46.2%)	2 (22.2%)	0.39	0.26	0.26	Fisher	0.88	1.00	0
Coronary Artery Disease (CAD)	binary	1	14 (53.8%)	7 (77.8%)							
Chronic Kidney Disease (CKD)	binary	0	20 (76.9%)	6 (66.7%)	0.87	0.66	0.66	Fisher	0.90	1.00	0
Chronic Kidney Disease (CKD)	binary	1	6 (23.1%)	3 (33.3%)							
Chronic Obstructive Pulmonary Disease (COPD)	binary	0	25 (96.2%)	8 (88.9%)	1.00	0.45	0.45	Fisher	0.88	1.00	0
Chronic Obstructive Pulmonary Disease (COPD)	binary	1	1 (3.8%)	1 (11.1%)							
Peripheral Arterial Disease (PAD)	binary	0	23 (88.5%)	8 (88.9%)	1.00	1.00	1.00	Fisher	1.00	1.00	0
Peripheral Arterial Disease (PAD)	binary	1	3 (11.5%)	1 (11.1%)							
Acetylsalicylic Acid (ASA use)	binary	0	10 (38.5%)	2 (22.2%)	0.63	0.45	0.45	Fisher	0.88	1.00	0
Acetylsalicylic Acid (ASA use)	binary	1	16 (61.5%)	7 (77.8%)							
Cilostazol / Pentoxifylline	binary	0	25 (96.2%)	8 (88.9%)	1.00	0.45	0.45	Fisher	0.88	1.00	0
Cilostazol / Pentoxifylline	binary	1	1 (3.8%)	1 (11.1%)							
NOAH	binary	0	25 (96.2%)	7 (77.8%)	0.31	0.16	0.16	Fisher	0.88	1.00	0
NOAH	binary	1	1 (3.8%)	2 (22.2%)							
Computed Tomography (CT)	binary	0	21 (80.8%)	2 (22.2%)	0.01	<0.01	<0.01	Fisher	0.09	0.09	0

Computed Tomography (CT)	binary	1	5 (19.2%)	7 (77.8%)							
Magnetic Resonance Imaging (MRI)	binary	0	20 (76.9%)	5 (55.6%)	0.43	0.39	0.39	Fisher	0.88	1.00	0
Magnetic Resonance Imaging (MRI)	binary	1	6 (23.1%)	4 (44.4%)							
Peripheral Angiography	binary	0	10 (38.5%)	4 (44.4%)	1.00	1.00	1.00	Fisher	1.00	1.00	0
Peripheral Angiography	binary	1	16 (61.5%)	5 (55.6%)							
Necrotizing Fasciitis	binary	0	19 (73.1%)	8 (88.9%)	0.61	0.65	0.65	Fisher	0.90	1.00	0
Necrotizing Fasciitis	binary	1	7 (26.9%)	1 (11.1%)							
Diabetic Nephropathy	binary	0	22 (84.6%)	4 (44.4%)	0.05	0.03	0.03	Fisher	0.29	0.81	0
Diabetic Nephropathy	binary	1	4 (15.4%)	5 (55.6%)							
Diabetic Retinopathy	binary	0	20 (76.9%)	6 (66.7%)	0.87	0.66	0.66	Fisher	0.90	1.00	0
Diabetic Retinopathy	binary	1	6 (23.1%)	3 (33.3%)							
Dialysis Status	binary	0	25 (96.2%)	8 (88.9%)	1.00	0.45	0.45	Fisher	0.88	1.00	0
Dialysis Status	binary	1	1 (3.8%)	1 (11.1%)							
Negative Pressure Wound Therapy	binary	0	21 (80.8%)	6 (66.7%)	0.68	0.40	0.40	Fisher	0.88	1.00	0
Negative Pressure Wound Therapy	binary	1	5 (19.2%)	3 (33.3%)							

Physiotherapy	binary	0	3 (11.5%)	5 (55.6%)	0.02	0.02	0.02	Fisher	0.22	0.42	0
Physiotherapy	binary	1	23 (88.5%)	4 (44.4%)							

Supplementary Table S5. Coding dictionary for categorical and ord. variables used in Datasets 1–2, reporting the processed variable name, variable type, numeric code, and the corresponding English category label.

Feature	Variable type	Code	Meaning / category label
ASA Physical Status Classification	ord.	1	ASA I
ASA Physical Status Classification	ord.	2	ASA II
ASA Physical Status Classification	ord.	3	ASA III
ASA Physical Status Classification	ord.	4	ASA IV
ASA Physical Status Classification	ord.	5	ASA V
Acetylsalicylic Acid (ASA use)	binary (nominal)	0	No
Acetylsalicylic Acid (ASA use)	binary (nominal)	1	Yes
Alcohol Consumption	binary (nominal)	0	No
Alcohol Consumption	binary (nominal)	1	Yes
Peripheral Angiography Success	ord. (4-level)	0	No angiography / not applicable
Peripheral Angiography Success	ord. (4-level)	1	Normal anatomy
Peripheral Angiography Success	ord. (4-level)	2	Unsuccessful
Peripheral Angiography Success	ord. (4-level)	3	Successful
Coronary Artery Disease (CAD)	binary (nominal)	0	No
Coronary Artery Disease (CAD)	binary (nominal)	1	Yes
Chronic Kidney Disease (CKD)	binary (nominal)	0	No
Chronic Kidney Disease (CKD)	binary (nominal)	1	Yes
Chronic Obstructive Pulmonary Disease (COPD)	binary (nominal)	0	No
Chronic Obstructive Pulmonary Disease (COPD)	binary (nominal)	1	Yes
CRP LRINEC	ord. (derived)	0	CRP < 150
CRP LRINEC	ord. (derived)	4	CRP ≥ 150

Computed Tomography (CT)	binary (nominal)	0	No
Computed Tomography (CT)	binary (nominal)	1	Yes
Cilostazol / Pentoxifylline	binary (nominal)	0	No
Cilostazol / Pentoxifylline	binary (nominal)	1	Yes
Contralateral Limb Status	ord. (5-level)	0	Normal
Contralateral Limb Status	ord. (5-level)	1	Forefoot amputation
Contralateral Limb Status	ord. (5-level)	2	Minor amputation
Contralateral Limb Status	ord. (5-level)	3	Below-knee amputation
Contralateral Limb Status	ord. (5-level)	4	Other/rare category (check raw entry)
Diabetic Nephropathy	binary (nominal)	0	No
Diabetic Nephropathy	binary (nominal)	1	Yes
Diabetic Retinopathy	binary (nominal)	0	No
Diabetic Retinopathy	binary (nominal)	1	Yes
Dialysis Status	binary (nominal)	0	No
Dialysis Status	binary (nominal)	1	Yes
Educational Status	ord.	0	No education
Educational Status	ord.	1	Literate only
Educational Status	ord.	2	Primary school
Educational Status	ord.	3	Middle school
Educational Status	ord.	4	High school
Educational Status	ord.	5	University
FPG LRINEC	ord. (derived)	0	FPG $\leq$ 180 mg/dL
FPG LRINEC	ord. (derived)	1	FPG > 180 mg/dL
Family History	binary (nominal)	0	No
Family History	binary (nominal)	1	Yes
Type of Footwear	binary (nominal)	0	Inappropriate
Type of Footwear	binary (nominal)	1	Appropriate
Hypertension (HT)	binary (nominal)	0	No

Hypertension (HT)	binary (nominal)	1	Yes
Hb LRINEC	ord. (derived)	0	Hb ≥ 13.5
Hb LRINEC	ord. (derived)	1	11 ≤ Hb < 13.5
Hb LRINEC	ord. (derived)	2	Hb < 11
Insulin Use	binary (nominal)	0	No
Insulin Use	binary (nominal)	1	Yes
Creatinine LRINEC	ord. (derived)	0	Creatinine ≤ 1.6
Creatinine LRINEC	ord. (derived)	2	Creatinine > 1.6
Magnetic Resonance Imaging (MRI)	binary (nominal)	0	No
Magnetic Resonance Imaging (MRI)	binary (nominal)	1	Yes
MRI Osteomyelitis Presence	ord. (4-level)	0	Not performed / No MRI data
MRI Osteomyelitis Presence	ord. (4-level)	1	Osteomyelitis absent
MRI Osteomyelitis Presence	ord. (4-level)	2	Other/unspecified (check raw entry)
MRI Osteomyelitis Presence	ord. (4-level)	3	Osteomyelitis present
Marital Status	binary (nominal)	0	Single
Marital Status	binary (nominal)	1	Married
Ambulation Status	binary (nominal)	0	No mobilization / non-ambulatory
Ambulation Status	binary (nominal)	1	Mobilization present / ambulatory
Necrotizing Fasciitis	binary (nominal)	0	No
Necrotizing Fasciitis	binary (nominal)	1	Yes
NOAH	binary (nominal)	0	No
NOAH	binary (nominal)	1	Yes
Negative Pressure Wound Therapy	binary (nominal)	0	No
Negative Pressure Wound Therapy	binary (nominal)	1	Yes
Na LRINEC	ord. (derived)	0	Na ≥ 135
Na LRINEC	ord. (derived)	2	Na < 135
Neuropathy Severity	ord.	0	No neuropathy
Neuropathy Severity	ord.	1	Mild
Neuropathy Severity	ord.	2	Moderate

Neuropathy Severity	ord.	3	Severe
Oral Antidiabetic Drug (OAD) Use	binary (nominal)	0	No
Oral Antidiabetic Drug (OAD) Use	binary (nominal)	1	Yes
Peripheral Arterial Disease (PAD)	binary (nominal)	0	No
Peripheral Arterial Disease (PAD)	binary (nominal)	1	Yes
PEDIS Classification	ord.	1	Grade 1
PEDIS Classification	ord.	2	Grade 2
PEDIS Classification	ord.	3	Grade 3
PEDIS Classification	ord.	4	Grade 4
Peripheral Angiography	binary (nominal)	0	No
Peripheral Angiography	binary (nominal)	1	Yes
Physiotherapy	binary (nominal)	0	No
Physiotherapy	binary (nominal)	1	Yes
Place of Residence	binary (nominal)	0	Rural
Place of Residence	binary (nominal)	1	Urban
Scintigraphy Osteomyelitis Presence	ord. (3-level)	0	Not performed / No scintigraphy data
Scintigraphy Osteomyelitis Presence	ord. (3-level)	1	Osteomyelitis absent
Scintigraphy Osteomyelitis Presence	ord. (3-level)	3	Osteomyelitis present
Sex	binary (nominal)	0	Male
Sex	binary (nominal)	1	Female
Smoking Status	binary (nominal)	0	No
Smoking Status	binary (nominal)	1	Yes
Tissue Culture	ord. (4-level)	0	Not recorded / not performed
Tissue Culture	ord. (4-level)	1	No growth
Tissue Culture	ord. (4-level)	2	Sensitive (Susceptible)
Tissue Culture	ord. (4-level)	3	Resistant
WBC LRINEC	ord. (derived)	0	WBC < 15
WBC LRINEC	ord. (derived)	1	15 ≤ WBC < 25
WBC LRINEC	ord. (derived)	2	WBC > 25
Wagner Classification	ord.	1	Grade 1

Wagner Classification	ord.	2	Grade 2
Wagner Classification	ord.	3	Grade 3
Wagner Classification	ord.	4	Grade 4
Wagner Classification	ord.	5	Grade 5

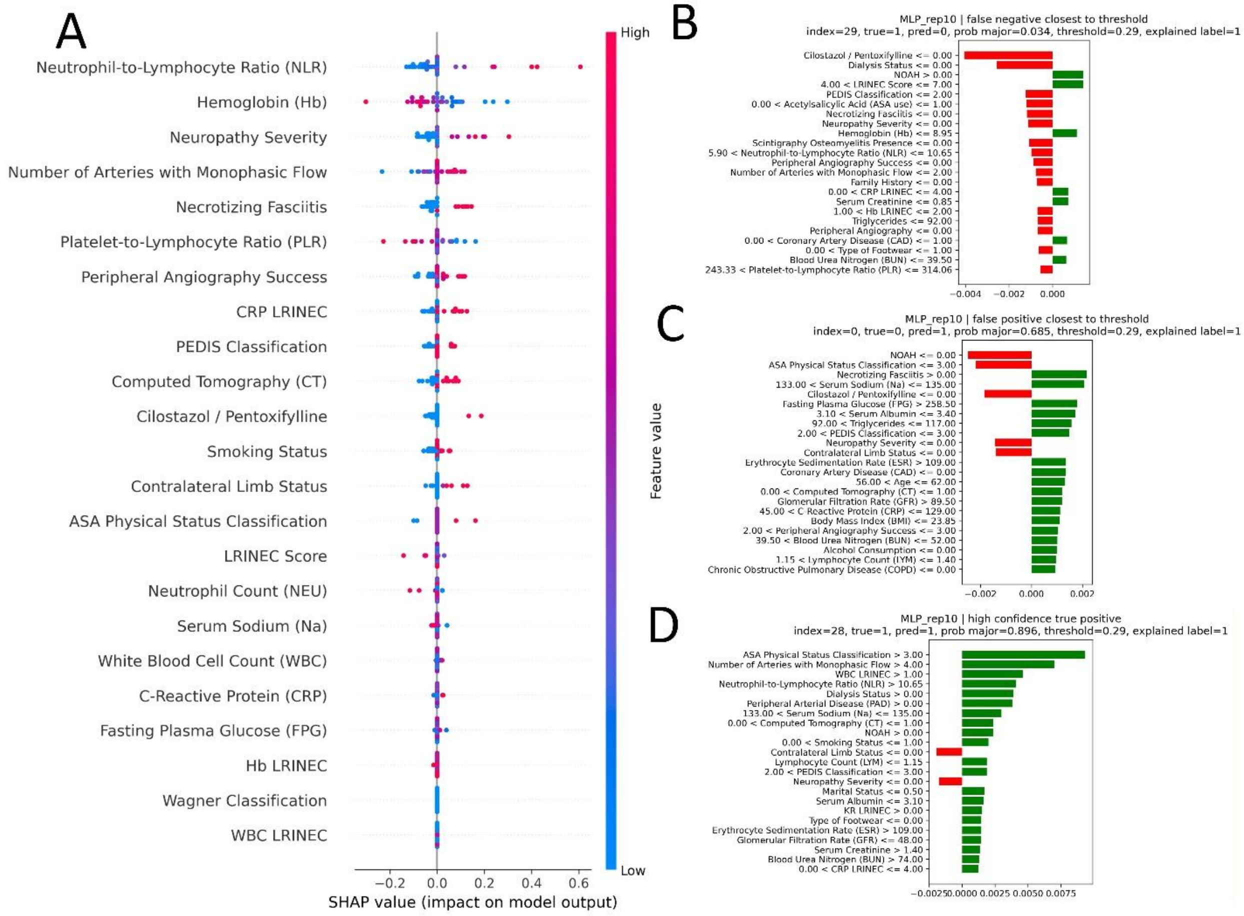

Supplementary Figure S1. Explainable artificial intelligence outputs for the MLP model from repetition 11 in the external cohort. A. SHAP beeswarm plot showing the global contribution of the selected features to the model output. B. LIME explanation for the false negative case closest to the decision threshold. C. LIME explanation for the false positive case closest to the decision threshold. D. LIME explanation for a high-confidence true positive case.
