# Supplementary material for: Machine learning–based prediction of major amputation risk after initial limb-preserving surgery in diabetic foot
Source: Front Endocrinol (Lausanne). 2026 Aug 3;17:1889329. doi: 10.3389/fendo.2026.1889329 (PMC13478262; doi:10.3389/fendo.2026.1889329)
Supplement: Supplementary file 1 [file DataSheet1.zip › supplementary material 2 1-2.pdf]

## Backend Architecture and Database Design

### 1. Web Application Architecture

The clinical decision support system was implemented as a prototype web application using the Django framework (v5.2) with a modular backend architecture. The system enables clinicians to upload patient data, obtain amputation risk predictions from multiple machine learning models simultaneously, and provide outcome feedback to support continuous model evaluation. The overall backend architecture is illustrated in Supplementary Figure S1.

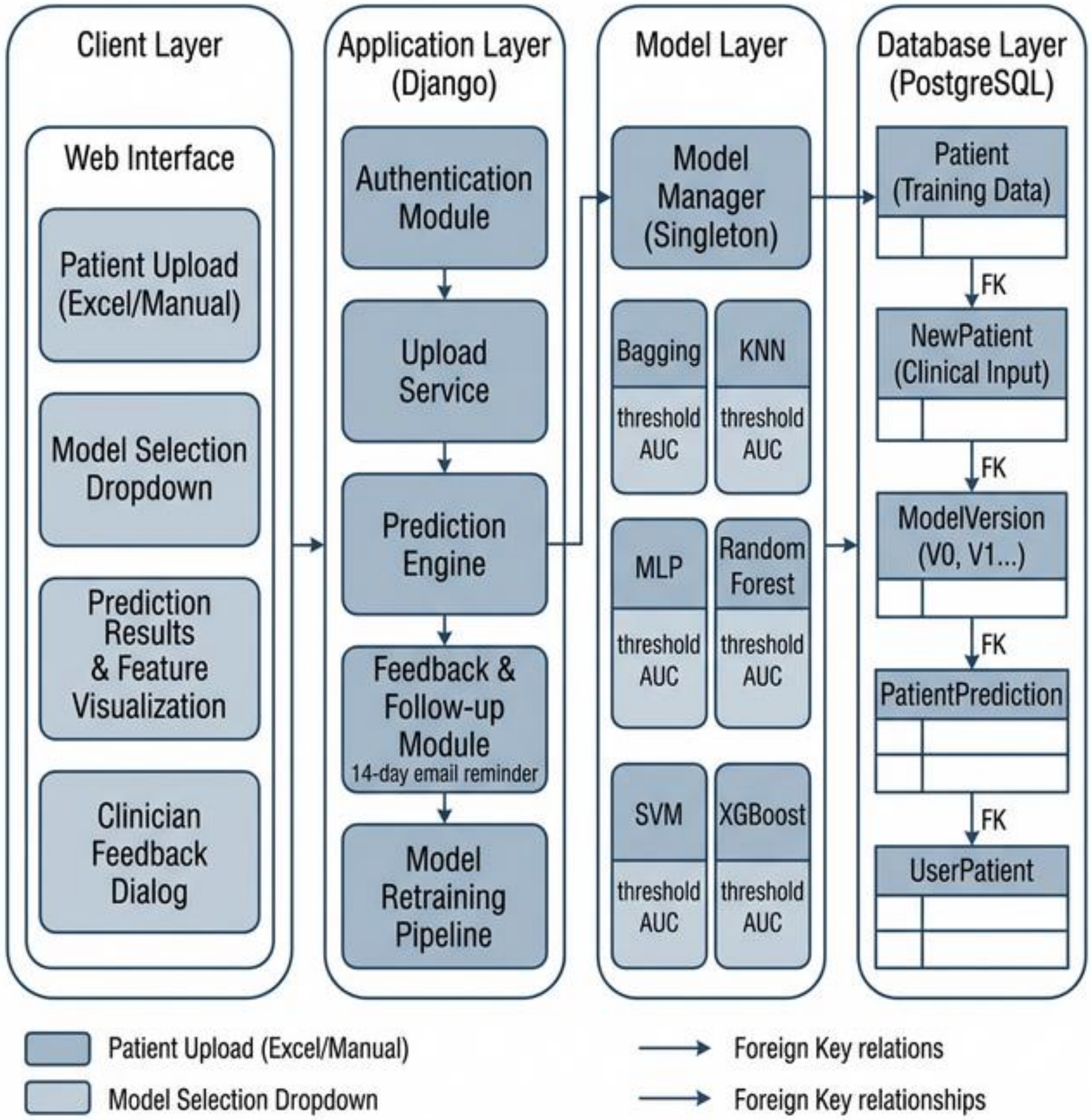


Supplementary Figure S1. System architecture of the clinical decision support web application.

The application follows a service-oriented design comprising four functional layers: request handling, model inference, visualization/projection support, and data persistence. The request handling layer processes two input modalities, single-patient XLSX file uploads and manual form entries through Django views that validate input, persist patient records, and delegate to the inference layer. The model layer maintains six concurrently loaded classification pipelines, each versioned in

the database. The visualization/projection support layer prepares two-dimensional feature-pair scatter plots from the fixed deployment subset to allow contextual comparison between the new patient and the historical cohort.

### Multi-Model Prediction Pipeline

The prediction subsystem employs a singleton Model Manager that loads all six trained classification pipelines (Bagging, KNN, MLP, Random Forest, SVM, and XGBoost) into memory upon application startup. Each pipeline encapsulates a complete inference workflow, including feature scaling, feature selection, and the final classifier stored as serialized scikit-learn Pipeline objects.

When a clinician uploads patient data, the system first persists the record in the NewPatient table without performing any prediction. The clinician then initiates concurrent inference across all six deployed classifiers from the interface, which triggers asynchronous prediction requests for all models. Each pipeline processes the same 23 input features and produces a binary classification (minor vs. major amputation) using its own optimized decision threshold derived via F2-score maximization on the hold-out validation set, subject to a minimum recall constraint of 0.70. In the deployed prototype, these 23 input features were fixed according to the common subset retained in repetition 11, and the input forms, stored pipelines, and database-facing deployment schema were configured around this shared subset. The prediction result, along with the associated confidence score, defined as the predicted probability of the assigned class is stored in the PatientPrediction table, linked to both the patient record and the specific model version. For the patient-level summary shown in the dashboard and stored in the NewPatient table, the system designates as the primary model the classifier with the highest confidence among the six model outputs; the corresponding predicted label is recorded as the patient-level prediction, and clinician feedback is collected with respect to this primary-model output.

To support visual contextualization, the frontend renders interactive two-dimensional scatter plots based on user-selected feature pairs from the 23-feature deployment subset, allowing the new patient to be compared against the historical cohort.

### Database Scheme

The relational database scheme was designed to support model versioning, per-model prediction tracking, and clinician feedback collection across the following core entities:

**Supplementary Table S1**. The relational database scheme

| Entity | Purpose | Key Fields |
|---|---|---|
| **Patient** | Deployment training records within the fixed 23-feature schema | 23 clinical features, label (0 = minor, 1 = major) |
| **NewPatient** | Clinician-submitted inference cases | 23 features, primary-model predicted_label (highest-confidence), user_feedback, final_label |
| **ModelVersion** | Model registry with versioning | model_name, version (V0, V1, …), threshold, AUC scores, is_active |
| **PatientPrediction** | Per-model prediction log | FK→NewPatient, FK→ModelVersion, predicted_label, confidence |

| | | |
|---|---|---|
| **UserPatient** | User–patient ownership | FK→User, FK→NewPatient, created_at |

The ModelVersion table enables systematic tracking of model iterations. Each version stores the optimized decision threshold and both internal (cross-validation) and external (hold-out) AUC-ROC scores, allowing administrators to compare model performance across retraining cycles. The is_active flag controls which model versions are exposed to clinicians, facilitating staged rollouts of updated models.

### Clinician Feedback and Follow-up Mechanism

Upon receiving a prediction, the clinician is prompted to assess its correctness through a modal dialog, with three options: correct, incorrect, or unsure. This feedback is recorded at the patient level in the NewPatient table, and when the clinician confirms or corrects the prediction, a final_label is derived accordingly.

For cases marked as unsure, typically when the clinical outcome is not yet determined, the architecture was designed to support an automated follow-up mechanism. A scheduled task can be configured to identify patients with pending feedback whose initial assessment occurred beyond a defined interval, and to dispatch reminder notifications to the responsible clinician, prompting re-evaluation. This mechanism is intended to ensure that outcome labels are eventually collected for the majority of cases, supporting future model retraining with clinician-verified ground truth.

### Model Retraining and Version Management

The platform architecture supports iterative model improvement through a structured retraining pipeline. When a sufficient number of clinician-verified cases accumulate in the NewPatient table, an administrator can initiate retraining. Verified cases (those with a non-null final_label) are merged with the existing training cohort in the Patient table. Within the deployed system, retraining is performed on the fixed 23-feature input schema derived from the repetition-11 deployment subset rather than by reconstructing the full 66-feature exploratory workflow used in the research phase. The six classification models are then retrained using the expanded dataset, and the resulting pipelines are registered in the ModelVersion table as a new version (e.g., V1). Previous versions remain accessible in the database for audit and comparison purposes, while the is_active flag is updated to route predictions through the latest models.

## 2. Web Based Decision Support Interface

The user interface of the developed clinical decision support system was implemented as a React.js based Single Page Application. This architecture enables user interactions to proceed without full page reloads and provides a more continuous and integrated workflow. The interface communicates with the Django REST API backend through RESTful HTTP requests, while authentication and session continuity are managed through a server-side cookie mechanism. Together with CSRF protection and authorization controls, this design ensures that only authenticated users can access protected components of the platform.

Upon first access, users are guided through a sequential onboarding flow consisting of two components, as illustrated in Supplementary Figure S2 and Supplementary Figure S3. The first component is a legal disclaimer screen (Supplementary Figure S2), which explicitly states that the

platform is intended for educational and research use, is not FDA approved, and should not be interpreted as a source of medical advice, diagnosis, or treatment recommendation. The disclaimer also emphasizes algorithmic limitations and requires the user to actively confirm acceptance before proceeding. The second component is an interactive tutorial screen (Supplementary Figure S3), which introduces the main workflow of the system through a three-step guided structure supported by visual demonstrations and numbered instructions. In the illustrated tutorial flow, the user is first shown how to download the Excel template, prepare the data according to the expected coding rules, upload the completed file, and initiate model-based analysis. The remaining tutorial steps then guide the user through result exploration and interface navigation. The onboarding state is preserved through cookies so that returning users are not repeatedly shown these screens unless the relevant cookie state is cleared or expires.

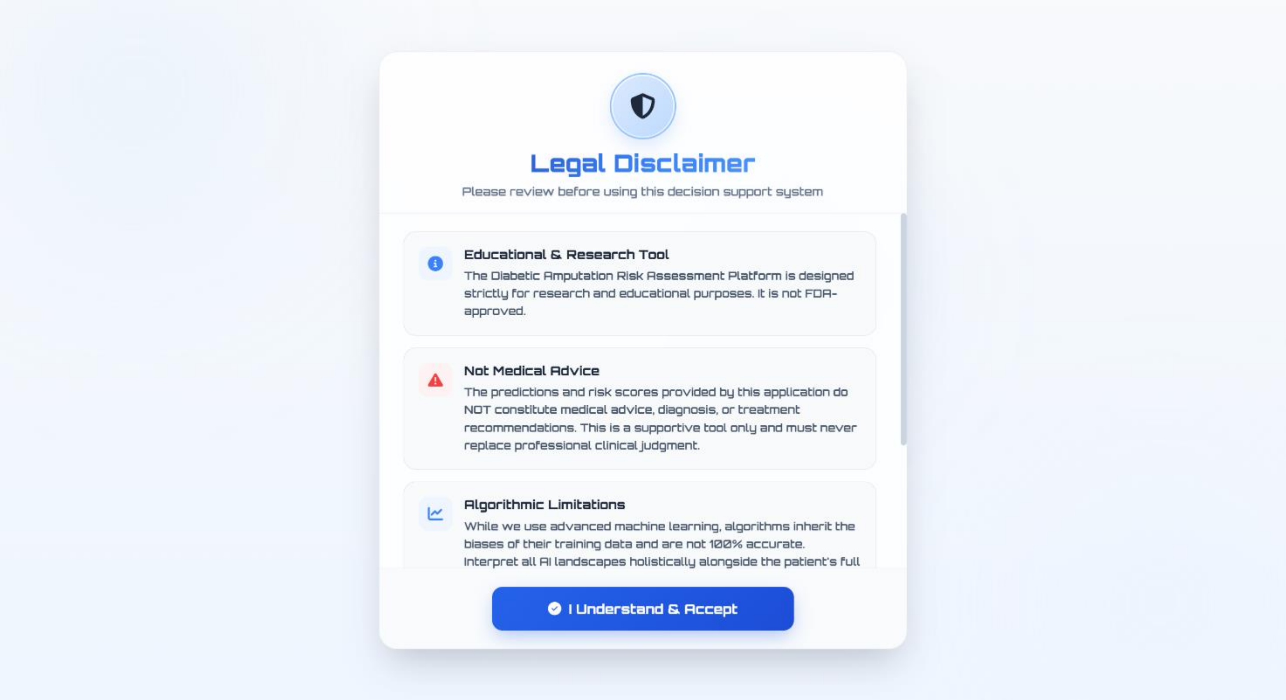


**Supplementary Figure S2.** Legal disclaimer screen requiring explicit acceptance before use.

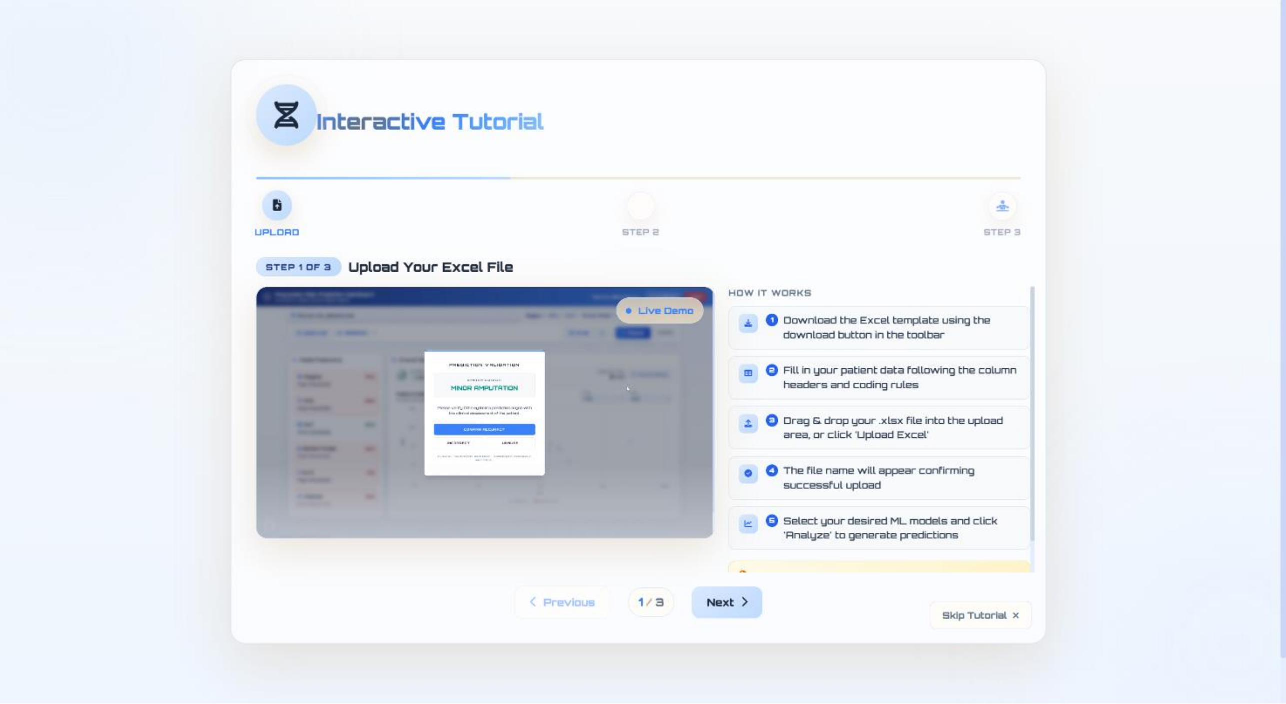


**Supplementary Figure S3.** Interactive tutorial with a three-step guided walkthrough

Access to the system is provided through an email- and password-based login mechanism, as presented in Supplementary Figure S4. The authentication page includes dedicated input fields for email address and password, a password visibility control, and a direct account creation link for new users. After successful authentication, a user session is initiated and the authentication state is maintained throughout the application via a centralized context-based structure. This provides a functional basis for secure navigation across protected views and consistent tracking of user state during the session.

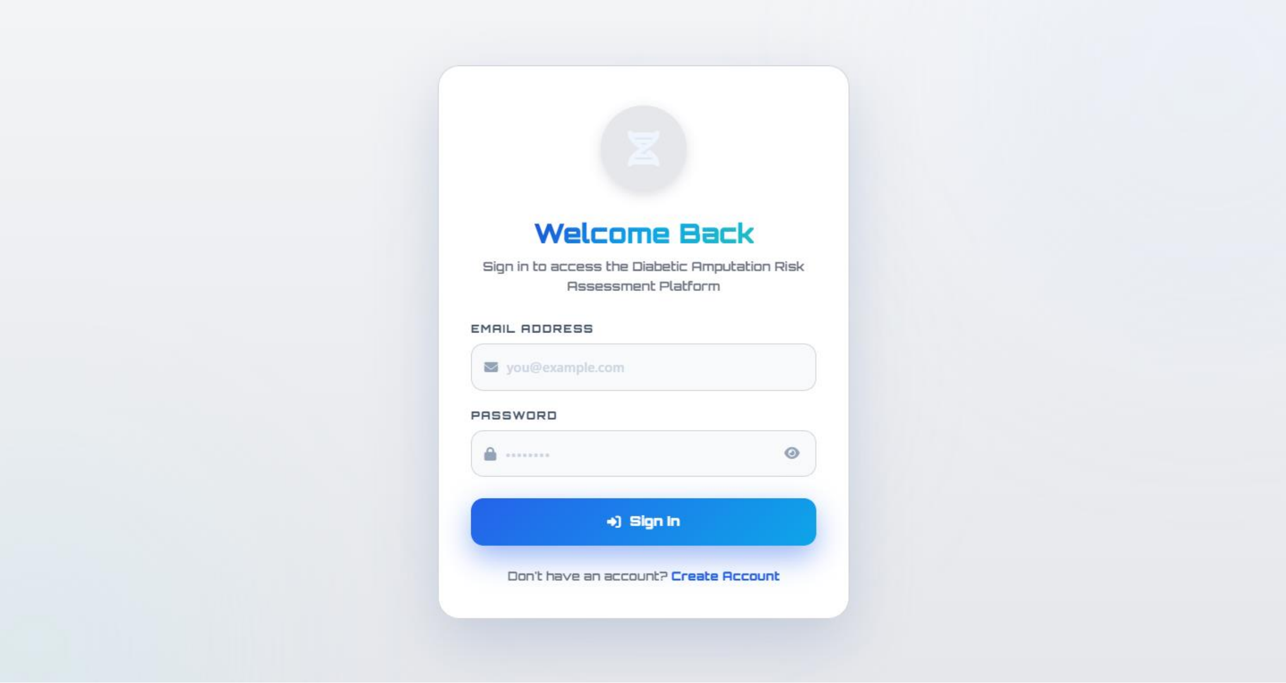


**Supplementary Figure S4.** User interaction flow of the Diabetic Amputation Risk Assessment Platform: authentication screen

The main dashboard as the central workspace of the system is given in Supplementary Figure S5. Before analysis, the left side prediction panel remains empty and the overall risk assessment area displays a placeholder state indicating that no patient has yet been analysed. At the same time, the upper control area provides the complete action set required to run the workflow, including Excel upload, model comparison display, manual patient entry, template download, analysis initiation, and reset functions. The model display area is implemented as a chip-based comparison interface in which Bagging, KNN, MLP, Random Forest, SVM, and XGBoost are presented together for simultaneous side-by-side review. In this way, the user can compare the outputs of the full six-model set on the same patient record within a single session. Once a file is uploaded, the source file name is displayed in the interface, thereby making the active analysis context explicit.

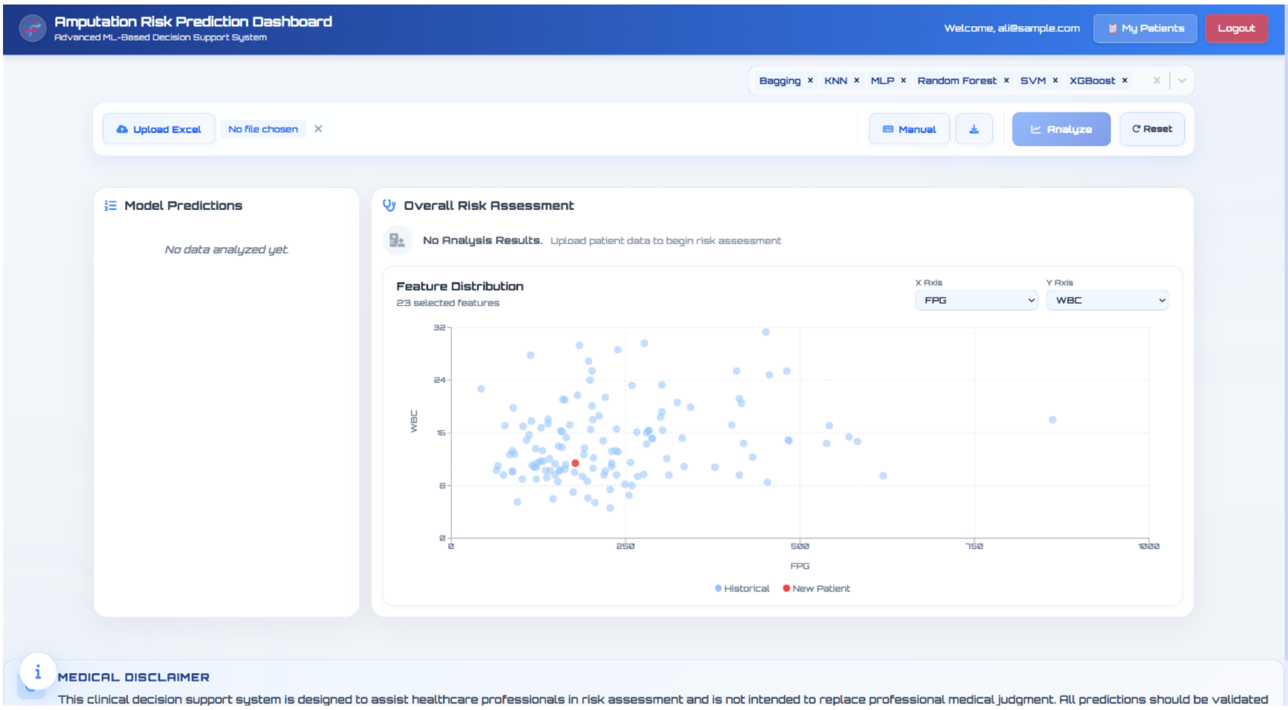


**Supplementary Figure S5.** User interaction flow of the Diabetic Amputation Risk Assessment Platform: dashboard before analysis

As an alternative to spreadsheet-based batch upload, a structured manual patient entry window was developed for controlled entry of individual patient records (Supplementary Figure S6-S8). The manual workflow is organized as a three-step form containing 23 selected clinical features. The first step, labeled Lab and Vitals, includes variables such as smoking status, fasting plasma glucose, white blood cell count, hemoglobin, C reactive protein, serum sodium, neutrophil count, neutrophil to lymphocyte ratio, platelet to lymphocyte ratio, and related LRINEC components. The second step, labeled Clinical Findings, captures structured examination and intervention related variables including medication history, contralateral limb status, number of arteries with monophasic flow, CT scan status, peripheral angiography success, necrotizing fasciitis, and neuropathy severity. The third step, labeled Scores, records summary scoring variables such as ASA score, Wagner classification, PEDIS classification, and LRINEC score. The wizard includes explicit step indicators, next step navigation, cancel controls, and utility actions such as Auto Fill and Clear All, thereby supporting both standardized data entry and rapid demonstration use.

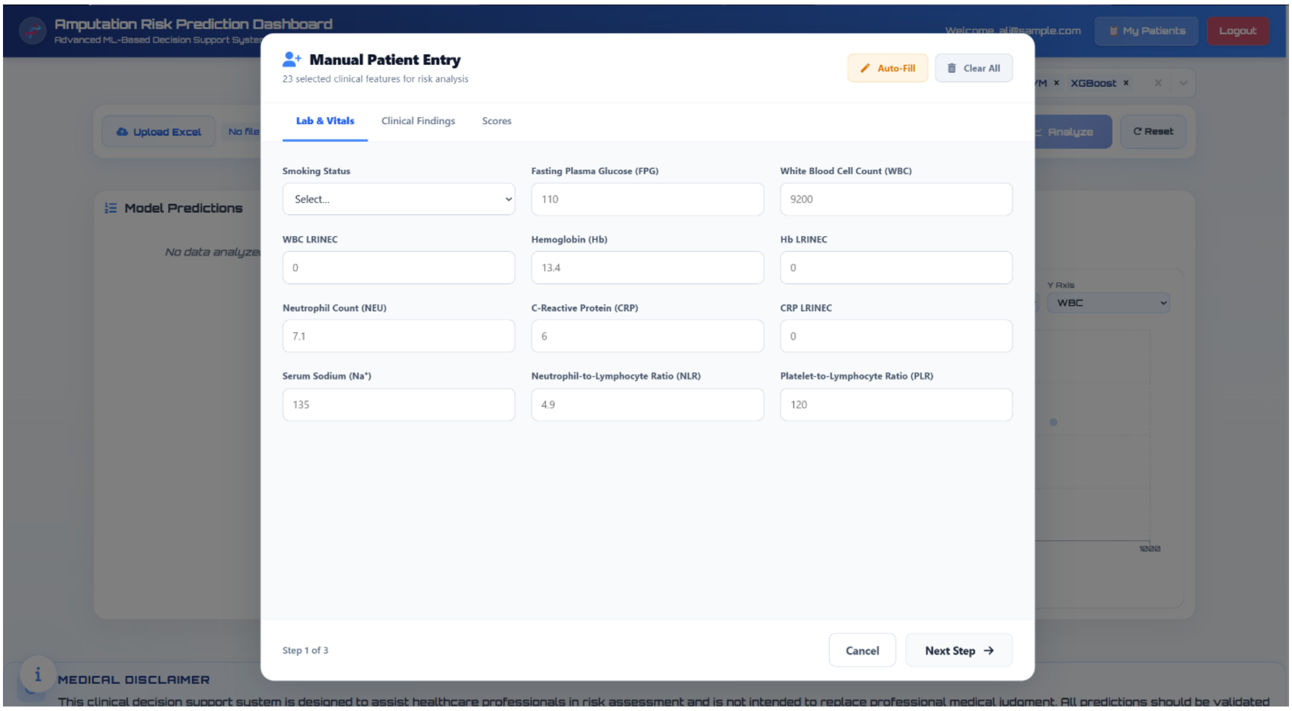


**Supplementary Figure S6.** User interaction flow of the Diabetic Amputation Risk Assessment Platform: multi step manual patient entry workflow covering Lab and Vitals.

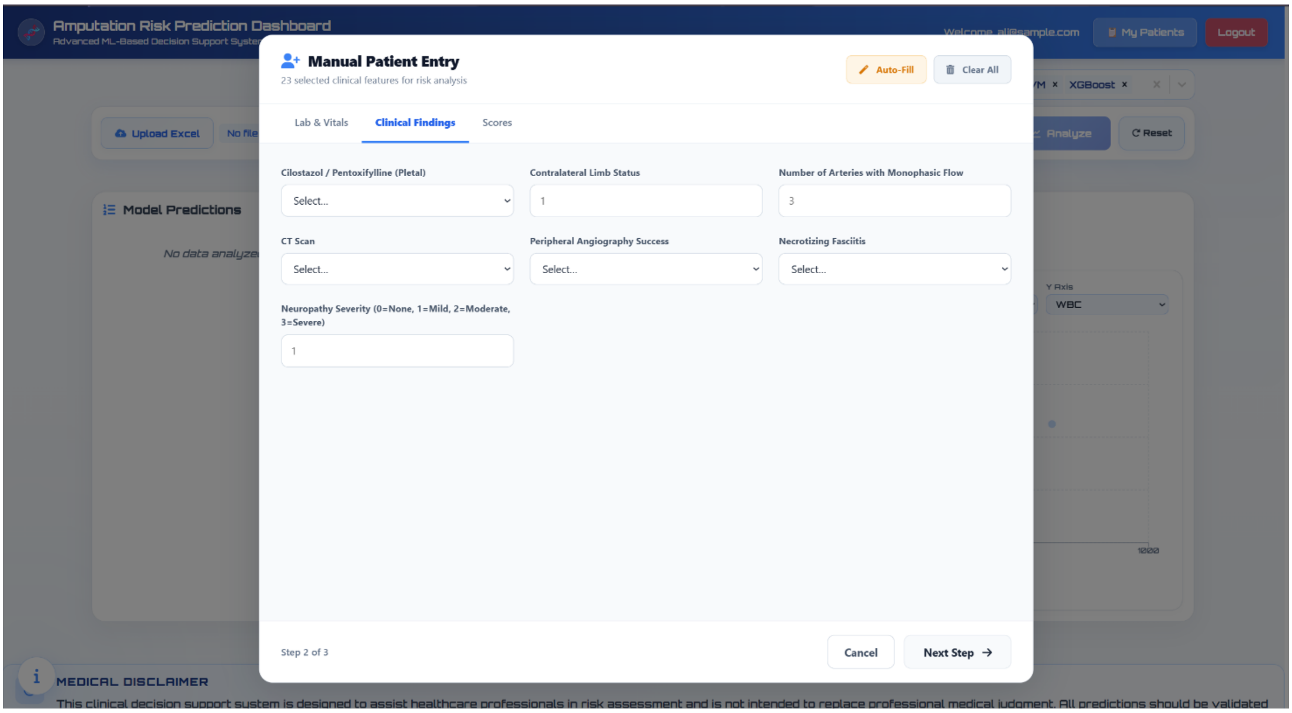


**Supplementary Figure S7.** User interaction flow of the Diabetic Amputation Risk Assessment Platform: multi step manual patient entry workflow covering Clinical Findings.

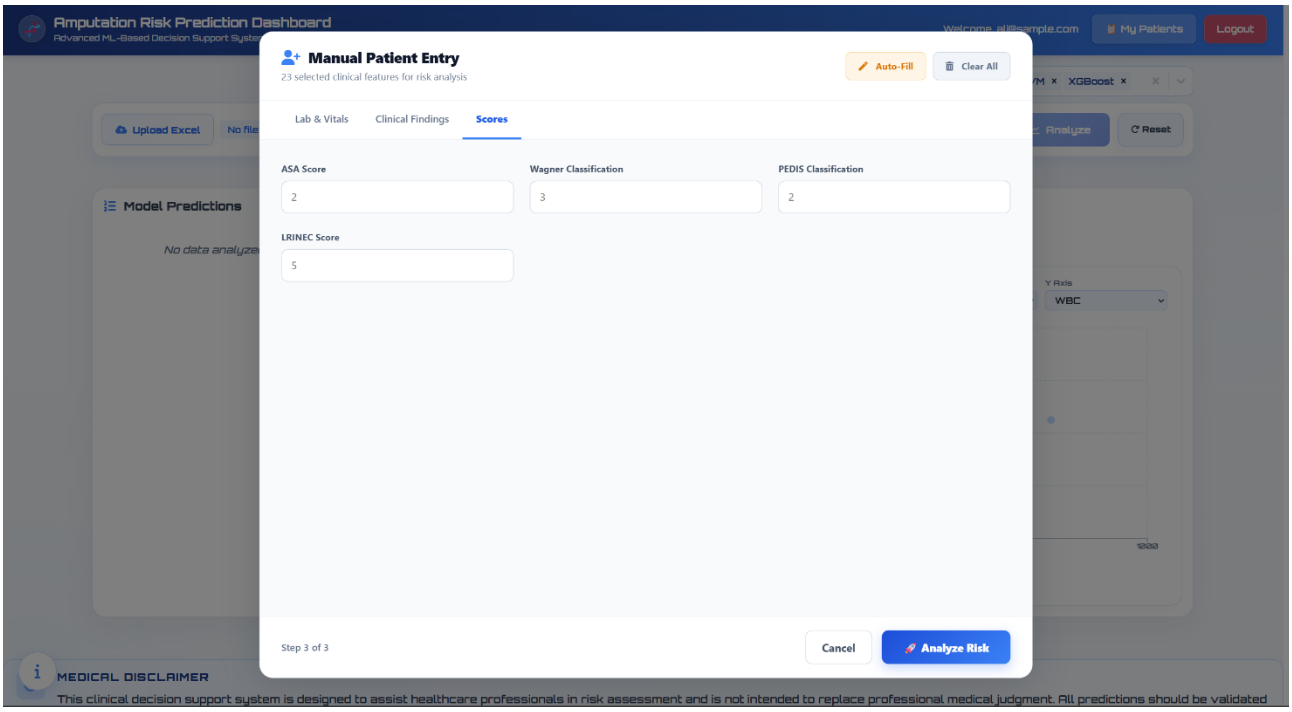


**Supplementary Figure S8.** User interaction flow of the Diabetic Amputation Risk Assessment Platform: Scores.

After the user initiates analysis, the system presents a prediction validation modal (Supplementary Figure S9-S10). In this window, the system output is displayed prominently and the clinician is asked to respond through one of three actions: Confirm Accuracy, Incorrect, or Unsure. This feedback mechanism provides a practical validation layer that can support later auditing, performance monitoring, and error analysis. The inclusion of an Unsure option is particularly valuable for representing situations in which the clinician does not wish to make a binary acceptance or rejection decision.

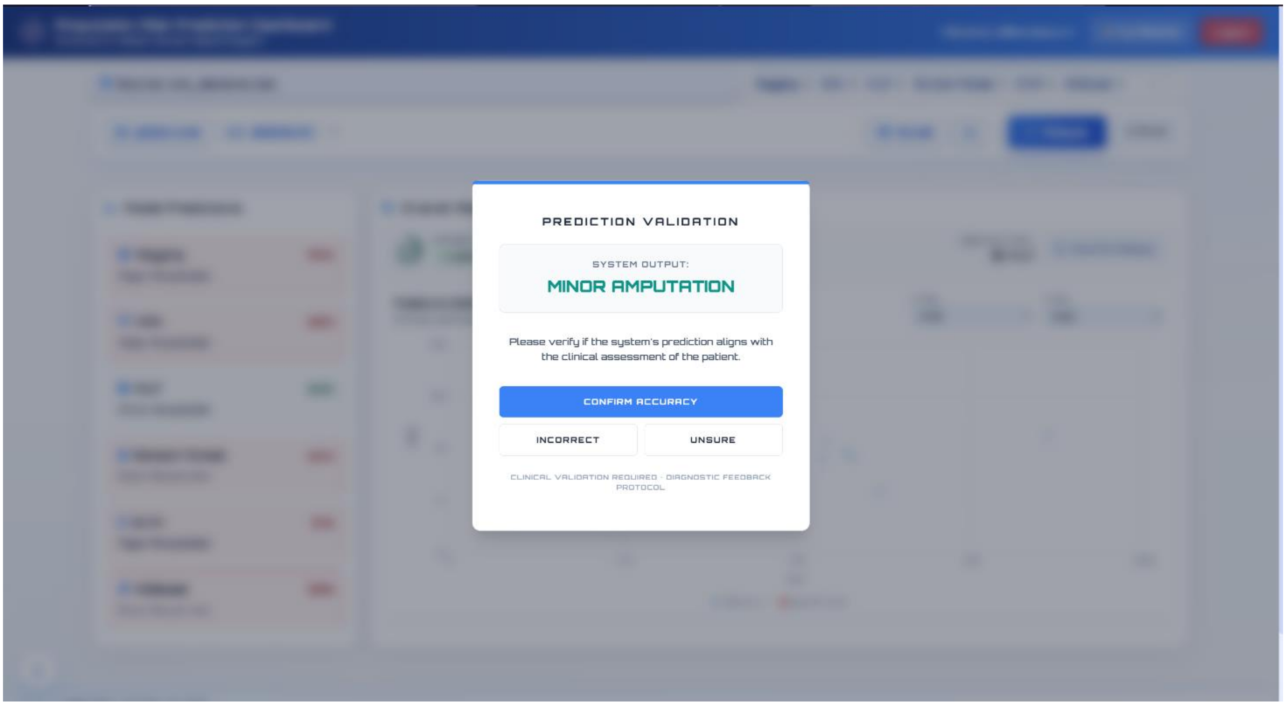

**Supplementary Figure S9.** User interaction flow of the Diabetic Amputation Risk Assessment Platform: prediction validation modal

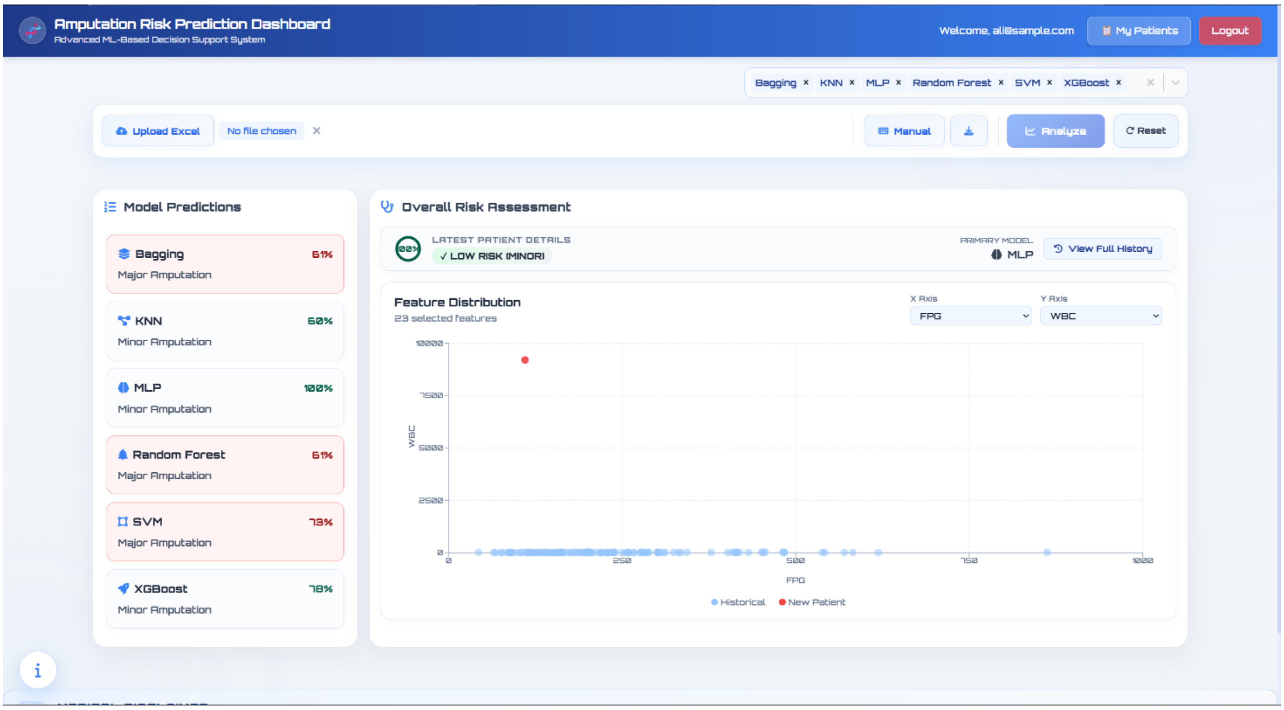


**Supplementary Figure S10.** User interaction flow of the Diabetic Amputation Risk Assessment Platform: post analysis dashboard with multi model predictions and feature distribution plot.

Following validation, the analyzed case is displayed within the post analysis dashboard view (Supplementary Figure S10). The left panel lists the selected models together with their predicted amputation class and associated confidence percentages. The right panel provides an overall risk assessment area containing the latest patient status, the highest-confidence risk label, the primary model reference, and a shortcut to the full patient history. In this summary view, the displayed risk label is taken from the model with the highest confidence score among the six concurrently generated predictions. Below this summary, a two-dimensional feature distribution plot places historical patients in blue and the newly analyzed patient in red. The user can dynamically assign the variables displayed on the x and y axes, allowing the current patient to be visually contextualized with respect to the previously analyzed population. This plot is intended as a supportive visual comparison component rather than a standalone explainability mechanism.

The system also includes a patient history management panel for reviewing previously analysed records (Supplementary Figure S11). This screen reports total saved cases together with summarized counts for high risk and low risk outcomes, and supports text-based search, category-based filtering, record refresh, and expandable row level inspection. Each record includes patient identifier information, selected summary variables, analysis outcome badges, validation status, save date, and deletion controls. This history structure enables users to revisit prior analyses, inspect their outcomes, and manage stored records directly within the same interface.

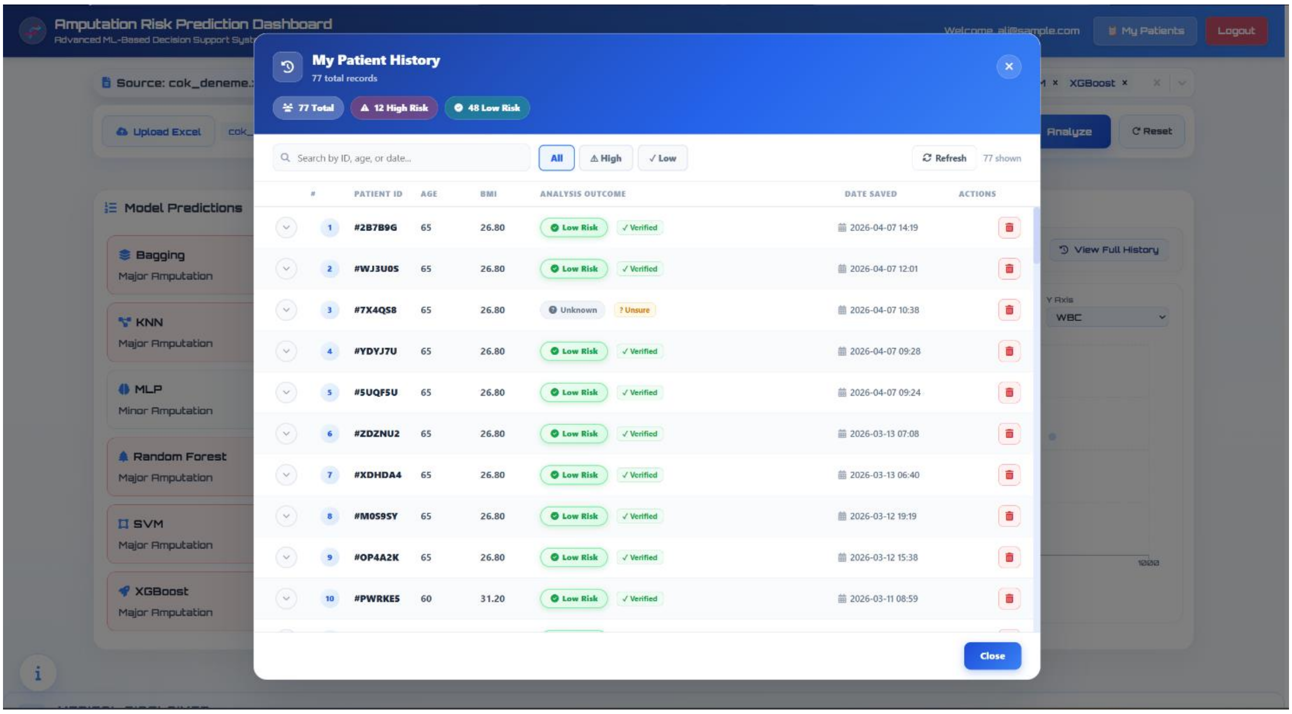


**Supplementary Figure S11.** User interaction flow of the Diabetic Amputation Risk Assessment Platform: patient history management panel.

Across the interface, readability, consistency, and ease of use were prioritized. A clean blue and white visual language was adopted across the dashboard, modal windows, and onboarding screens, while risk categories were encoded consistently, with high risk emphasized in red and low risk in green. In addition, the platform repeatedly communicates that it is a research-oriented decision support tool and should not be used as a standalone substitute for clinical judgment.
